# Demographic Characteristics of Participants in Rheumatoid Arthritis Randomized Clinical Trials: A Systematic Review

**DOI:** 10.1001/jamanetworkopen.2019.14745

**Published:** 2019-11-13

**Authors:** Adrienne Strait, Francine Castillo, Sonam Choden, Jing Li, Evans Whitaker, Titilola Falasinnu, Gabriela Schmajuk, Jinoos Yazdany

**Affiliations:** 1 School of Medicine, University of California, San Francisco; 2 Division of Rheumatology, University of California San Francisco VA Medical Center, San Francisco; 3 Division of Rheumatology, University of California, San Francisco; 4 Medical Library, University of California, San Francisco; 5 Department of Health Research and Policy, Stanford School of Medicine, Stanford, California

## Abstract

**Importance:**

Racial/ethnic minority groups, women, and elderly people experience a disproportionate burden of disease in rheumatoid arthritis (RA), making it particularly important to examine drug therapies in these populations. Despite a national health agenda to improve representation of diverse populations in randomized clinical trials (RCTs), there have been few large-scale analyses examining RCT demographic characteristics within rheumatology and none focusing on RA.

**Objective:**

To characterize the representation of racial/ethnic minority groups, women, and elderly people through a comprehensive systematic review of RA RCTs.

**Data Sources:**

A literature search of PubMed’s MEDLINE database was conducted to identify RA RCTs in adults 19 years and older published in English between January 1, 2008, and January 1, 2018.

**Study Selection:**

Randomized double-blind RCTs examining any systemic, disease-modifying therapy were included. Secondary analyses of previously published RCTs were excluded. Of 1195 identified records, 240 articles (20.1%) met final selection criteria. The analysis focused on RCTs with at least 1 US-based site.

**Data Extraction and Synthesis:**

Data were extracted and synthesized according to the PRISMA guidelines for systematic reviews. Studies were screened for eligibility criteria. Demographic data on the age, sex, and race/ethnicity of RCT participants were extracted. Data analysis was conducted from October 25, 2018, to March 15, 2019.

**Main Outcomes and Measures:**

Representation of race/ethnicity and sex, defined as the proportion of total participants that belonged to each racial/ethnic group or sex. Trends in proportions over time were examined and compared with US demographic data.

**Results:**

A total of 240 RCTs with 77 071 participants were included. Of 126 RCTs with at least 1 US-based site (52.5%), the enrollment of minority racial/ethnic groups was significantly lower than their representation within the US Census population (16% vs 40%; *P* < .001), and the enrollment of men was significantly lower than the incidence of RA in men nationally (20.4% vs 28.6%; *P* < .001). There was no trend toward improved representation of racial/ethnic minority groups or men over time.

**Conclusions and Relevance:**

Given the disproportionate burden of RA among racial/ethnic minority groups, it is imperative that policy makers better incentivize the inclusion of racial/ethnic minority groups in RA RCTs.

## Key Points

**Question:**

Is there equitable representation of racial/ethnic minority groups, women, and elderly people within rheumatoid arthritis (RA) randomized clinical trials (RCTs)?

**Findings:**

In this systematic review of 240 RA RCTs including 77 071 participants, there was significant underrepresentation of racial/ethnic groups throughout 2008 to 2018 with no trend toward improved representation of minority racial/ethnic groups in US-based RCTs during the 10-year period. Additionally, men were underrepresented in US-based RA RCTs, and elderly people were often excluded from RA RCTs.

**Meaning:**

Given the disproportionate burden of RA in racial/ethnic minority groups, it is imperative that policy makers better incentivize their inclusion in RA RCTs.

## Teaser

This systematic review examines the representation in rheumatoid arthritis randomized clinical trials of racial/ethnic minority groups compared with the representation of these populations in the United States and the representation of women and elderly people with the prevalence of rheumatoid arthritis among these groups nationally.

## Introduction

Rheumatoid arthritis (RA) is associated with significant morbidity and mortality, resulting in substantial health care utilization and cost. Approximately 1.5 million people in the United States have RA, accounting for 0.6% of the adult population.^1^ Racial/ethnic minority groups, women, and elderly people experience a disproportionate burden of RA, making it particularly important to examine therapies in these populations.^2,3,4,5,6,7,8,9^

Increasing the proportion of underrepresented groups in randomized clinical trials (RCTs) is a critical component of eliminating health disparities and is a priority of the US national health agenda. The 1993 National Institutes of Health (NIH) Revitalization Act^10^ requires minority groups to be included in all NIH-funded clinical research unless a justification is approved by the NIH. A 2001 policy^10^ mandates that proposals for NIH-defined phase III RCTs create processes for identifying differences in treatment responses among racial/ethnic groups if the intervention effect is expected to vary among them. A 2017 NIH policy revision^11^ requires that applicable NIH-defined phase III clinical trials submit valid subgroup analyses by race/ethnicity and sex/gender to ClinicalTrials.gov.^12^

Randomized clinical trial representation is particularly relevant to RA given the rapidly changing treatment landscape. The development of novel biologic disease-modifying antirheumatic drugs (DMARDs) has been accompanied by rapid increase in RA RCTs examining their efficacy, with new treatment guidelines placing an emphasis on the early and aggressive use of these drugs.^13,14^ Meanwhile, emerging evidence suggests that race and ethnicity may influence an individual’s response to DMARD therapy, and several studies have indicated that new RA therapies may be less effective in racial/ethnic minority groups.^5,15^

Despite the importance of clinical trial representation to the national health agenda, there have been few large-scale analyses examining the diversity of clinical trial participants within rheumatology and none about RA, to our knowledge. The objective of this study was to perform a systematic review of RA RCTs published over a 10-year period from January 1, 2008, to January 1, 2018, to evaluate whether there was adequate representation of racial/ethnic minority groups, women, and elderly people.

## Methods

This study was conducted in accordance with the Preferred Reporting Items for Systematic Reviews and Meta-analyses (PRISMA) reporting guidelines with the exception of those relevant only to meta-analyses (eg, risk of bias assessment). Institutional review board approval and informed consent were not obtained given that the study was a systematic review of the literature, per human subject regulations from the US Department of Health and Human services.^16^

### Search Strategy

With consultation from a professional research librarian (E.W.), we developed a search strategy to identify RA RCTs that were published in MEDLINE between January 1, 2008, and January 1, 2018. We used “Rheumatoid Arthritis” and “humans” as Medical Subject Headings (MeSH) terms and “randomized controlled trial” as a publication type to generate the following search phrase: “arthritis, rheumatoid” (MeSH) AND (“2008/01/01” [PDAT]: “2018/01/01” [PDAT] AND “humans” [MeSH Terms]) AND (“randomized controlled trial” [ptyp] AND “humans” [MeSH Term]). Filters were applied to identify English-language studies in adults 19 years and older. Our search was limited to RCTs published within a 10-year period, since there was a tremendous and unprecedented increase in RA RCTs during this period, allowing us to examine a very large sample size. The English-language filter was applied to increase the likelihood that studies would have at least 1 US-based site.

**Figure 1.**
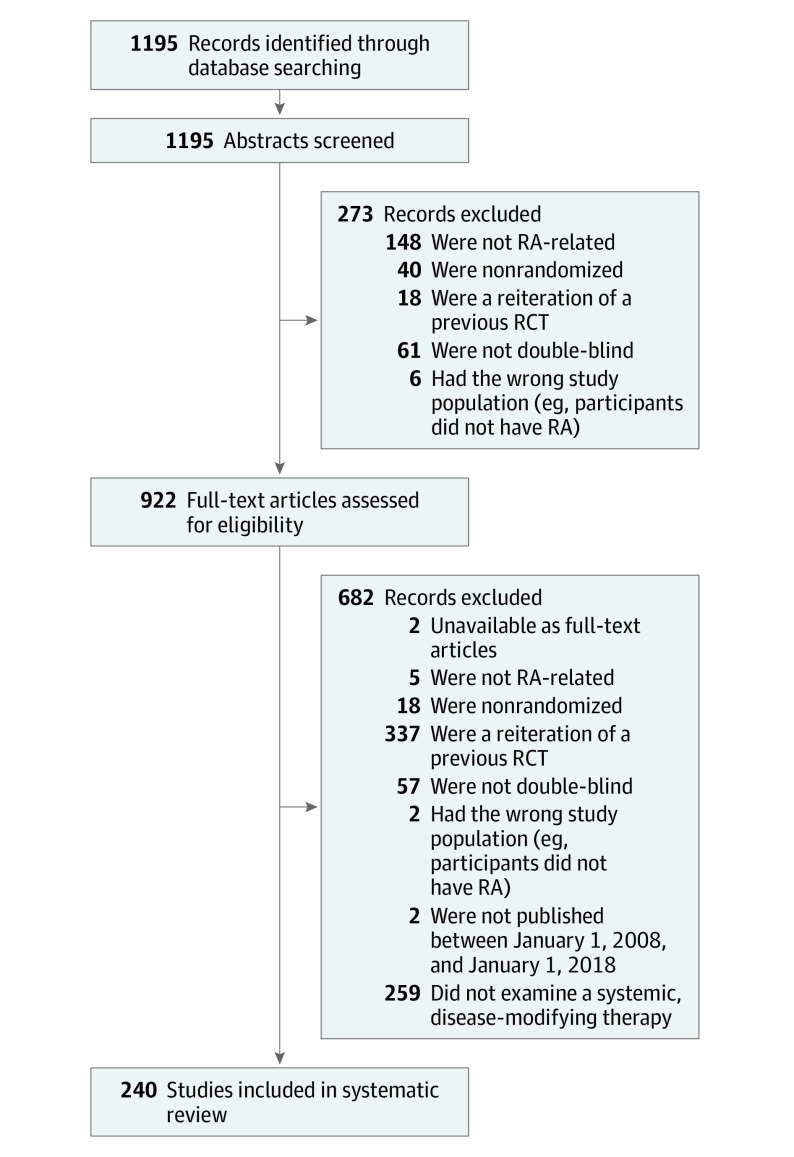
Flow Diagram for Selection of Studies RA indicates rheumatoid arthritis; RCT, randomized clinical trial.

Final search results were saved to EndNote reference management software version X8.2 (Clarivate Analytics). Randomized clinical trials were included if (1) they were double-blinded, (2) participants were adults with RA, and (3) they examined a systemic, disease-modifying therapy. We excluded studies that involved reiterations or secondary analyses of previously published RCTs. Abstracts were independently screened for inclusion by 2 reviewers (A.S. and F.C.) using Rayyan (Qatar Computing Research Institute), a systematic review web-based application. There was complete agreement between reviewers; therefore, adjudication by a third party was not required. A total of 273 studies were excluded during the abstract screening phase, leaving 922 trials for full-text review (Figure 1). Full-text articles were screened for eligibility by at least 1 reviewer (A.S., F.C., or S.C.) using a process adapted from a previously published systematic review,^17^ and another 682 trials were excluded.

### Outcome Reporting

In addition to general study characteristics, we extracted demographic data on participant age, sex, and race/ethnicity. If these data were not found within the published article or associated supplemental information, reported clinical trial registry numbers were used to identify the information through the US National Library of Medicine clinical trial registry website.^12^ Race/ethnicity reporting was based on US Census Bureau guidelines,^18^ with race including the categories American Indian or Alaska Native, Asian or Native Hawaiian or other Pacific Islander, black, white, and other; ethnicity included the categories Hispanic or Latino and not Hispanic or Latino. To account for the fact that not all studies followed these guidelines, we extracted data on the way in which race/ethnicity was reported (eg, race reported and Hispanic ethnicity not mentioned, only ethnicity reported). All studies reporting any race/ethnicity data were included in final analyses.

### Statistical Analysis

The analysis of race/ethnicity and sex representation was limited to RCTs with at least 1 US-based site, since we were most interested in US trends. Representation for each racial/ethnic group was calculated as a weighted mean for each year: the denominator was the total number of participants among all studies with at least 1 US-based site that reported racial/ethnic composition, and the numerator was the number of participants reported in the racial/ethnic group. A separate denominator of US-only sites was used for some of the analyses. Representation of men was calculated in a similar way, with the total number of participants among all studies with at least 1 US-based site that reported sex demographics as the denominator.

We used χ^2^ tests to compare (1) representation across racial/ethnic groups with the overall demographics of the United States using the Census Bureau estimates for 2013 to 2017,^19^ (2) representation of men in RA RCTs with the overall age-adjusted prevalence of RA in men (RA is approximately 2.5-fold as prevalent in women as in men),^6^ (3) trends in nonwhite representation vs white representation over time, and (4) trends in male representation vs female representation over time. Analyses were performed using Stata statistical software version 15 (StataCorp). *P* values were 2-tailed, and *P* values less than .05 were used as the criterion for statistical significance for all analyses. Data analysis was conducted from October 25, 2018, to March 15, 2019.

## Results

### Search Retrieval Results

After application of the exclusion criteria, 240 RCTs with 77 071 participants were included in the review (Figure 1; eAppendix in the Supplement). Our analysis focused on 126 RCTs (52.5%) with at least 1 US-based site. Within this subgroup, 119 RCTs (94.4%) were industry-funded, 99 RCTS (78.6%) enrolled 200 or more participants, and 119 RCTs (94.4%) investigated a biologic or synthetic DMARD (Table). There was no statistically significant difference in the percentage of RCTs in this subgroup that reported race or ethnicity compared with the total sample (64.3% vs 54.2%; *P* = .06). Of RCTs that reported race or ethnic demographic data, 57 (45.2%) reported race but not Hispanic ethnicity. Forty-five RCTs (35.7%) did not report on race/ethnicity.

**Table.** Characteristics of the Rheumatoid Arthritis RCTs Included in the Systematic Review

Characteristic	RCTs, No. (%)	
	Total (N = 240)	With ≥1 US-Based Site (n = 126)
Sex inclusion		
Both	231 (96.3)	124 (98.4)
Women only	3 (1.3)	0
Not reported	6 (2.5)	2 (1.6)
Age inclusion		
Had exclusion criteria for any upper age limit	99 (41.3)	38 (30.2)
Exclusions by upper age limit, y		
>100 or >99	3 (1.3)	3 (2.4)
>85 or >80	21 (8.8)	11 (8.7)
>75 or >74	43 (17.9)	16 (12.7)
>70 or >69	14 (5.8)	4 (3.2)
>65, >64, or >60	18 (17.5)	4 (3.2)
Reported mean age and SD	193 (80.4)	96 (76.2)
Race/ethnicity reporting		
Race reported and Hispanic ethnicity not reported	86 (35.8)	57 (45.2)
Race reported and Hispanic reported as separate race	5 (2.1)	5 (4.0)
Race and ethnicity reported	12 (5.0)	11 (8.7)
Only ethnicity reported	27 (11.3)	8 (6.3)
Neither race nor ethnicity reported	110 (45.8)	45 (35.7)
Funding source		
Industry	205 (85.4)	119 (94.4)
Nonindustry	19 (7.9)	5 (4.0)
Jointly funded	7 (2.9)	2 (1.6)
Not stated	9 (3.8)	0
Total enrollment, participants		
≤50	32 (13.3)	7 (5.6)
51 to 100	28 (11.7)	8 (6.3)
101 to 200	37 (15.4)	12 (9.5)
≥200	143 (59.6)	99 (78.6)
Drug class being studied		
Biologic or new synthetic DMARD	214 (89.2)	119 (94.4)
Conventional DMARD	15 (6.3)	4 (3.2)
Glucocorticoid	6 (2.5)	2 (1.6)
Other	5 (2.1)	1 (0.8)

Abbreviations: DMARD, disease-modifying antirheumatic drug; RCT, randomized clinical trial.

### Race/Ethnicity Representation

#### Temporal Trends

**Figure 2.**
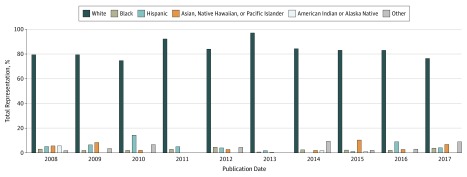
Trends in Racial/Ethnic Representation in Rheumatoid Arthritis Randomized Clinical Trials With at Least 1 US-Based Site

Figure 2 presents temporal trends in the proportion of included participants across racial/ethnic groups for RA RCTs with at least 1 US-based site. Overall, white participants were the most commonly represented, ranging from 74.6% in 2010 to 97.0% in 2013 (Figure 2). During the 10-year period, black representation ranged from 0.6% in 2013 to 4.7% in 2012; Hispanic representation ranged from 0% in 2014 to 14.4% in 2010; Asian, Native Hawaiian, and Pacific Islander representation ranged from 0% in 2011 to 10.4% in 2015; and American Indian or Alaska Native representation ranged from 0% in 2010, 2011, 2013, 2016, and 2017 to 5.7% in 2008. While there were fluctuations in the representation of different racial/ethnic groups over time, there were no significant trends in the representation of nonwhite groups vs white groups over the observed period (*P* for trend = .72).

#### Comparison to National Demographics

**Figure 3.**
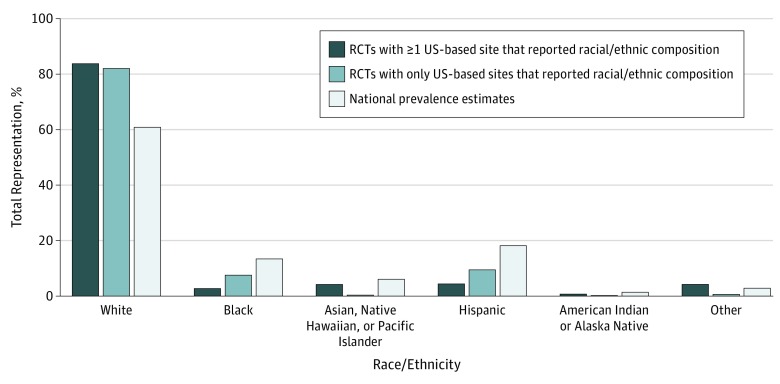
Trends in Racial/Ethnic Representation in Rheumatoid Arthritis Randomized Clinical Trials (RCTs) Compared With National Estimates National estimates were obtained from 2013 through 2017 Census Bureau data.

We compared the representation of race/ethnicity among RA RCTs with at least 1 US-based site to the overall representation of racial/ethnic groups within the US population (Figure 3). Of 126 RCTs with at least 1 US-based site (52.5%), the enrollment of minority racial/ethnic groups was significantly lower than their representation within the US census population (16% vs 40%; P < .001). For example, black participants represented 2.7% of enrollment in RA RCTs, which was significantly lower than the representation of this group nationally (13.4%) (*P* < .001). The representation of individuals in RA RCTs identifying as Hispanic was 4.4%, which was also significantly lower than the representation of this group nationally (18.1%) (*P* < .001). A comparable pattern was seen for the other racial/ethnic minority groups examined. We found similar differences when comparing the racial/ethnic enrollment in RCTs with US-only sites to national demographics (Figure 3).

### Sex Reporting and Representation

**Figure 4.**
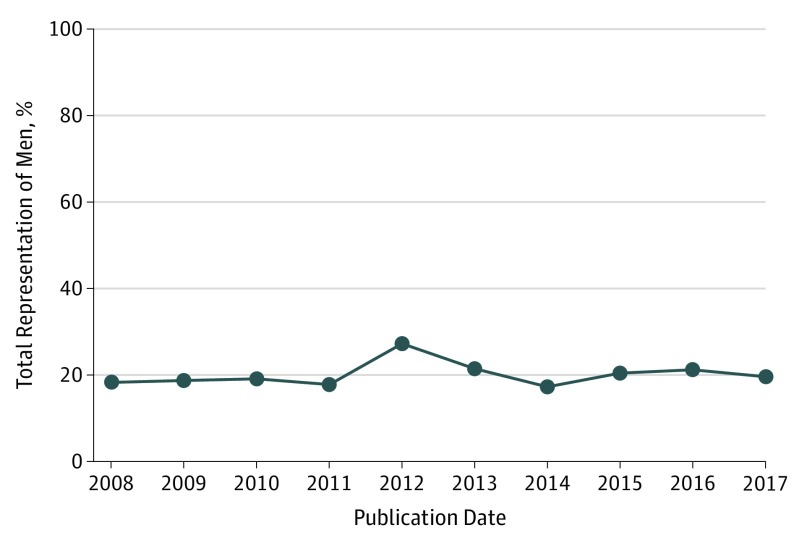
Trends in Male Representation in Rheumatoid Arthritis Randomized Clinical Trials With at Least 1 US-Based Site

Among 240 RCTs included in the review, 231 (96.3%) included both women and men, with 3 RCTs specifying the sole inclusion of women participants (Table). Figure 4 presents the temporal trends in male representation in RA RCTs with at least 1 US-based site. Over the 10-year period, male enrollment ranged from 17.3% in 2014 at its minimum to 27.3% in 2012 at its maximum. Overall, there was no significant trend in the enrollment of men vs women over time (*P* for trend = .22).

A comparison of the representation of men in US-based RA RCTs to the representation of men in RA cases nationally showed that the enrollment of men in RA RCTs with at least 1 US-based site was significantly lower than the burden of men with RA nationally (20.4% vs 28.6%; *P* < .001). The enrollment of men in RA RCTs with US-only sites was also significantly smaller than the representation of men with RA nationally (22.3% vs 28.6%; *P* < .001).

### Inclusion of Elderly Populations

An upper age limit was used as an exclusion criterion in 99 RA RCTs (41.3%) included in the review (Table). Randomized clinical trials most commonly excluded adults older than 74 or 75 years (43 RCTs [17.9%]). The subgroup of RCTs with at least 1 US-based site had a lower proportion of RCTs with exclusion criteria containing an upper age limit when compared with all included RCTs. Among 193 RCTs (80.4%) that reported age of the participants, the mean (SD) age of participants was 52.6 (11.4) years.

## Discussion

Despite national efforts to increase diversity in RCT participation, there was no trend toward improved representation of minority racial/ethnic groups in US-based RA RCTs over the examined period, with significant underrepresentation of these groups throughout. While racial and ethnic minority groups comprise approximately 40% of the US population,^19^ they only represented 16% of the population for RA RCTs. We found that the enrollment of men was significantly lower than the proportion of men with RA nationally,^6^ suggesting that men have been underrepresented in US-based RA RCTs during the examined 10-year period. Despite the large burden of disease among elderly people, we found that elderly people were often excluded from RA RCTs; in addition, the mean age of the RCT populations included in this review was lower than the mean age of people with RA estimated by population-based studies.^20^

The overall lack of minority representation in RA RCTs with US-based sites is consistent with systematic reviews examining racial/ethnic representation in US clinical trials for other diseases. For example, a 2004 systematic review^21^ of cancer trials found that Hispanic and black participants were markedly underrepresented. Systematic reviews examining participation in cardiovascular disease^22^ and smoking cessation^23^ RCTs found similar disparities in the enrollment of racial/ethnic minority groups. A 2018 systemic lupus erythematosus (SLE) review^17^ found that black and Asian participants were underrepresented in the enrollment of SLE RCTs, while participants identifying as Hispanic were overrepresented. This differs only slightly from our results, which showed that all nonwhite racial/ethnic groups were underrepresented in RCTs when compared to the prevalence of each within the US population.

In contrast to our findings, other reviews have identified significant changes in racial/ethnic representation over time.^17,21^ Of note, the periods examined by these studies differed from that in our review. For example, an SLE review of the period from 1997 to 2017^17^ found that time was associated with an increase in the representation of Asian, Hispanic, and Native American participants and a decrease in the representation of black participants after 2011. A 2004 review of racial/ethnic representation in National Cancer Institute cancer RCTs^21^ found that the representation of racial/ethnic minority groups decreased between 1995 and 2002.

The underrepresentation of men in RA RCTs is concordant with the SLE review,^17^ which showed men were consistently underenrolled in comparison to their representation in prevalence estimates for SLE. A 2019 systematic review and meta-analysis^24^ examining RA and osteoarthritis trial demographics found that both men and women were adequately represented. The review was not focused on US RCTs, which may explain the differences in our findings. Reviews in other medical specialties have found women to be underrepresented in RCTs.^25,26,27^

Our review also found that more than 40% of the RA RCTs published in the last 10 years excluded elderly participants. This is consistent with an emerging body of literature highlighting the underrepresentation of elderly participants in RCTs for a wide-ranging number of diseases. Of note, a 2019 systematic review and meta-analysis^24^ examining RA and osteoarthritis RCT participation also demonstrated underrepresentation of elderly participants.

### Race/Ethnicity Representation

There are many historic and systems-based factors that may affect the willingness of racial/ethnic minority groups to participate in clinical trials. The historical mistreatment of minority populations by medical researchers has led to distrust of the health care system.^28^ This is further compounded by current systems that perpetuate racial discrimination. In addition, studies have shown that attitudes toward treatment options and health beliefs differ among patients with RA by racial/ethnic group.^29,30^

If the RCTs in this review contained strict exclusion criteria that limited the participation of patients with comorbidities, this could potentially lead to higher rates of exclusion of certain racial/ethnic groups. For example, a 2018 systematic review^31^ examining the comorbidities limiting the participation of cancer patients in early phase RCTs found that most RCTs contained exclusion criteria restricting enrollment of the participants most likely to represent the populations treated in clinical settings.

Another explanation is perhaps that current policies are not effectively promoting RCT diversity. Most RA RCTs in this systematic review were industry-funded, suggesting that industry research may play a particularly large role in the identified underrepresentation. Current US Food and Drug Administration (FDA) regulations require sponsors of Investigational New Drug applications to report the race/ethnicity of RCT participants and summarize safety and effectiveness data by race/ethnicity; yet, in contrast to the NIH, there are no FDA policies mandating the inclusion of diverse populations.^32^ In addition, FDA regulations are focused on Investigational New Drugs and therefore do not apply to all industry-funded RCTs.

It would be beneficial for future RCTs to collect information about race/ethnicity at all stages of enrollment (ie, invitation, screening, randomization) to allow us to better target solutions. For example, if racial/ethnic minority groups are underrepresented among RCT invitees, then this would indicate that efforts are needed to expand outreach to include more diverse populations. In contrast, if a decrease in representation is seen after the invitation stage, this might suggest an unwillingness to participate or suboptimal recruitment procedures and would emphasize the importance of exploring solutions to the systemic factors described above. Another question of interest is whether the race/ethnicity of study investigators is associated with the diversity of invited participants.

Recent evidence that race and ethnicity may influence an individual's response to DMARD therapy underscores the need to increase representation of minority groups within RA clinical research. A retrospective study in the United Kingdom by Helliwell et al^15^ found that South Asian patients, as compared with Northern European patients, were more likely to stop DMARD therapy early because of lack of effectiveness and dermatological adverse effects. In another study by Greenberg et al,^5^ Hispanic patients had higher adjusted RA disease activity than white patients despite being treated with biologic drugs more frequently. While the goal of the 2017 NIH policy revision^11^ was to incentivize analyses of racial/ethnic differences in intervention effect, this policy does not counter all issues associated with underrepresentation because subgroup analyses will have low statistical power if minority populations have a small sample size. To address these gaps, effectiveness studies specific to racial/ethnic minority groups should be conducted to determine whether treatment responses to current RA therapies in these groups differ from white populations. The study by D’Cruz et al^33^ modeled this concept in SLE by examining the efficacy and safety of belimumab in black patients with SLE.

It was surprising that there was no trend toward improved inclusion of minority racial/ethnic groups in US-based RA RCTs between 2008 and 2017 despite the passage of the 1993 and 2001 NIH policies.^10^ One possible explanation is that increases in representation associated with these policies had tapered off prior to 2008. The 2017 NIH policy revision^11^ would not have affected representation trends in our review, given that it did not take effect until December 12, 2017. Future research could examine trends in racial/ethnic representation in RA RCTs in the several years before and after the passage of each NIH policy to evaluate impact.

Of note, 36% of RA RCTs with at least 1 US-based site did not include any race/ethnicity information. Some of this underreporting may have been a result of policy gaps; while the NIH and FDA have guidelines requiring reporting of study data by race/ethnicity, these do not encompass all types of trials. In contrast to our review, the SLE review^17^ of racial/ethnic representation identified a smaller proportion (9%) of RCTs with US-based sites that did not report racial/ethnic composition, suggesting that there may be factors specific to RA RCTs associated with underreporting.^17^ These factors were not evident from our review but suggest an opportunity for future exploration.

### Sex Representation

The underrepresentation of men in RA RCTs during the examined 10-year period was consistent with the findings of the systematic review in SLE^17^ but at odds with sex representation trends in other nonrheumatologic medical fields. This discrepancy may be explained by the widely publicized increased prevalence of rheumatic diseases in women, which may have affected their inclusion within RCTs for these diseases. In addition, a 2007 study^34^ suggested that women were more likely to participate in scientific research than their male counterparts. Given our findings, it may also be beneficial for rheumatology-specific campaigns to advocate for greater inclusion of men in RCTs in RA and SLE.

### Inclusion of Elderly Populations

The exclusion of elderly populations from many of the RA RCTs conducted in the last 10 years raises concerns about the generalizability of RA RCT results to older populations. This is of particular importance given evidence demonstrating that elderly populations respond differently to drug therapies than their younger counterparts as a result of increased comorbidities, polypharmacy, and changes in pharmacokinetics that accompany aging.^35,36^ Given the findings of this review, it may be necessary to conduct studies that include solely elderly populations to ascertain if current RA treatments are effective and well-tolerated in this group.

It is also vital that we enact policies that limit the formal exclusion of elderly participants from RCTs. The NIH formulated a revision to the Inclusion Across the Lifespan policy^37^ that took effect January 2019. It requires clinical research grantees to include individuals of all ages when conducting research supported by the NIH unless there are scientific or ethical reasons to exclude them and requires studies to provide data on participant age at enrollment. While we are optimistic that this new policy will encourage increased inclusion of elderly participants in RA RCTs, it will be important for future reviews to investigate its effect.

### Limitations

This study had limitations. Excluding RCTs that did not report race/ethnicity information may have biased our results toward the null; the excluded studies were likely to have lower rates of minority representation compared with the included studies. In addition, limiting our search to PubMed’s MEDLINE database and including only randomized double-blind trials may have led to a biased sample of higher-quality studies, suggesting that our findings may underestimate the magnitude of the problem. Future reviews could consider expanding their search to include other search engines and study types. Our review only included RCTs examining systemic, disease-modifying therapies, excluding a large number of RA RCTs that examined other types of interventions (eg, those testing integrative approaches, such as activity and diet coaching). In addition, our race/ethnicity analysis used population distribution estimates as a comparison point to approximate the expected burden of RA disease by race/ethnicity nationally, and we chose to limit our examination of race/ethnicity to the categories used by the US Census Bureau. Region-specific RA prevalence estimates are not yet available and therefore we do not have data to examine how RCT representation for specific geographic areas compares to the area-specific demographics. This consideration may be appropriate for future exploration.

This review was the first to simultaneously examine the representation of 3 demographic factors (race/ethnicity, sex, and age) within RA RCTs, to our knowledge. While a 2018 systematic review^24^ examined sex and age representation in RA and osteoarthritis RCTs, this is the first review to characterize racial/ethnic representation within RA RCTs. To our knowledge, only one other similar systematic review^17^ was conducted in the rheumatology literature, which analyzed the clinical trial representation of sex and racial/ethnic minority groups in SLE RCTs. Our review included a large number of RCTs, and final analyses focused on US-based RCTs, allowing for implications to be directly relevant to US policy and clinical research.

## Conclusion

Despite national efforts to increase diversity in RCT participation, there was no trend toward improved representation of racial/ethnic minority groups in RA RCTs with at least 1 US-based site between 2008 and 2018, and the enrollment of racial/ethnic minority groups was significantly lower than their representation within the US census population. Given the disproportionate burden of RA among racial/ethnic minority groups, it is imperative that policy makers better incentivize the inclusion of racial/ethnic minority populations in RA RCTs. This review also found that the enrollment of men in RA RCTs with at least 1 US-based site was significantly lower than the prevalence of RA in men nationally and that elderly people were often excluded from RA RCTS.

## Notes

Supplement. **eAppendix.** Bibliography of Included Articles
