# Supplementary material for: Demographic Characteristics of Participants in Rheumatoid Arthritis Randomized Clinical Trials: A Systematic Review
Source: JAMA Netw Open. 2019 Nov 13;2(11):e1914745. doi: 10.1001/jamanetworkopen.2019.14745 (PMC6902779; doi:10.1001/jamanetworkopen.2019.14745)
Supplement: Supplement. — eAppendix. Bibliography of Included Articles [file jamanetwopen-2-e1914745-s001.pdf]

Supplementary Online Content

Strait A, Castillo F, Choden S, et al. Demographic characteristics of participants in rheumatoid arthritis randomized clinical trials: a systematic review. *JAMA Netw Open*. 2019;2(11):e1914745. doi:10.1001/jamanetworkopen.2019.14745

eAppendix. Bibliography of Included Articles

This supplementary material has been provided by the authors to give readers additional information about their work.

© 2019 Strait A et al. *JAMA Network Open.*

eAppendix. Bibliography of Included Articles

1. Aira LE, Hernandez P, Prada D, et al. Immunological evaluation of rheumatoid arthritis patients treated with itolizumab. *mAbs.* 2016;8(1):187-195.
2. Aletaha D, Bingham CO, 3rd, Tanaka Y, et al. Efficacy and safety of sirukumab in patients with active rheumatoid arthritis refractory to anti-TNF therapy (SIRROUND-T): a randomised, double-blind, placebo-controlled, parallel-group, multinational, phase 3 study. *Lancet (London, England).* 2017;389(10075):1206-1217.
3. Alten R, Gomez-Reino J, Durez P, et al. Efficacy and safety of the human anti-IL-1beta monoclonal antibody canakinumab in rheumatoid arthritis: results of a 12-week, Phase II, dose-finding study. *BMC musculoskeletal disorders.* 2011;12:153.
4. Alten R, Gram H, Joosten LA, et al. The human anti-IL-1 beta monoclonal antibody ACZ885 is effective in joint inflammation models in mice and in a proof-of-concept study in patients with rheumatoid arthritis. *Arthritis research & therapy.* 2008;10(3):R67.
5. Alten RE, Zerbini C, Jeka S, et al. Efficacy and safety of pamapimod in patients with active rheumatoid arthritis receiving stable methotrexate therapy. *Annals of the rheumatic diseases.* 2010;69(2):364-367.
6. Atsumi T, Yamamoto K, Takeuchi T, et al. The first double-blind, randomised, parallel-group certolizumab pegol study in methotrexate-naive early rheumatoid arthritis patients with poor prognostic factors, C-OPERA, shows inhibition of radiographic progression. *Annals of the rheumatic diseases.* 2016;75(1):75-83.
7. Bae SC, Kim J, Choe JY, et al. A phase III, multicentre, randomised, double-blind, active-controlled, parallel-group trial comparing safety and efficacy of HD203, with innovator etanercept, in combination with methotrexate, in patients with rheumatoid arthritis: the HERA study. *Annals of the rheumatic diseases.* 2017;76(1):65-71.
8. Bakker MF, Jacobs JW, Welsing PM, et al. Low-dose prednisone inclusion in a methotrexate-based, tight control strategy for early rheumatoid arthritis: a randomized trial. *Annals of internal medicine.* 2012;156(5):329-339.
9. Baluom M, Samara E, Grossbard EB, Lau DT. Fostamatinib, a Syk-kinase inhibitor, does not affect methotrexate pharmacokinetics in patients with rheumatoid arthritis. *Journal of clinical pharmacology.* 2011;51(9):1310-1318.
10. Bao J, Yue T, Li T, He DY, Bao YX. Good response to infliximab in rheumatoid arthritis following failure of interleukin-1 receptor antagonist. *International journal of rheumatic diseases.* 2016;19(4):370-376.
11. Bao J, Yue T, Liu W, et al. Secondary failure to treatment with recombinant human IL-1 receptor antagonist in Chinese patients with rheumatoid arthritis. *Clinical rheumatology.* 2011;30(5):697-701.
12. Beals C, Baumgartner R, Peterfy C, et al. Magnetic resonance imaging of the hand and wrist in a randomized, double-blind, multicenter, placebo-controlled trial of infliximab for rheumatoid arthritis: Comparison of dynamic contrast enhanced assessments with semi-quantitative scoring. *PloS one.* 2017;12(12):e0187397.
13. Behrens F, Tak PP, Ostergaard M, et al. MOR103, a human monoclonal antibody to granulocyte-macrophage colony-stimulating factor, in the treatment of patients with moderate rheumatoid arthritis: results of a phase Ib/IIa randomised, double-blind, placebo-controlled, dose-escalation trial. *Annals of the rheumatic diseases.* 2015;74(6):1058-1064.
14. Bejarano V, Quinn M, Conaghan PG, et al. Effect of the early use of the anti-tumor necrosis factor adalimumab on the prevention of job loss in patients with early rheumatoid arthritis. *Arthritis and rheumatism.* 2008;59(10):1467-1474.
15. Bijlsma JWJ, Welsing PMJ, Woodworth TG, et al. Early rheumatoid arthritis treated with tocilizumab, methotrexate, or their combination (U-Act-Early): a multicentre, randomised, double-blind, double-dummy, strategy trial. *Lancet (London, England).* 2016;388(10042):343-355.
16. Blanco FJ, Moricke R, Dokoupilova E, et al. Secukinumab in Active Rheumatoid Arthritis: A Phase III Randomized, Double-Blind, Active Comparator- and Placebo-Controlled Study. *Arthritis & rheumatology (Hoboken, NJ).* 2017;69(6):1144-1153.
17. Boumans MJ, Houbiers JG, Verschueren P, et al. Safety, tolerability, pharmacokinetics, pharmacodynamics and efficacy of the monoclonal antibody ASK8007 blocking osteopontin in patients with rheumatoid arthritis: a randomised, placebo controlled, proof-of-concept study. *Annals of the rheumatic diseases.* 2012;71(2):180-185.
18. Boyle DL, Soma K, Hodge J, et al. The JAK inhibitor tofacitinib suppresses synovial JAK1-STAT signalling in rheumatoid arthritis. *Annals of the rheumatic diseases.* 2015;74(6):1311-1316.
19. Braun J, Kastner P, Flaxenberg P, et al. Comparison of the clinical efficacy and safety of subcutaneous versus oral administration of methotrexate in patients with active rheumatoid arthritis: results of a six-month, multicenter, randomized, double-blind, controlled, phase IV trial. *Arthritis and rheumatism.* 2008;58(1):73-81.
20. Briot K, Rouanet S, Schaeverbeke T, et al. The effect of tocilizumab on bone mineral density, serum levels of Dickkopf-1 and bone remodeling markers in patients with rheumatoid arthritis. *Joint, bone, spine : revue du rhumatisme.* 2015;82(2):109-115.

© 2019 Strait A et al. *JAMA Network Open.*

21. Bruyn GA, Tate G, Caeiro F, et al. Everolimus in patients with rheumatoid arthritis receiving concomitant methotrexate: a 3-month, double-blind, randomised, placebo-controlled, parallel-group, proof-of-concept study. *Annals of the rheumatic diseases.* 2008;67(8):1090-1095.
22. Burmester GR, Blanco R, Charles-Schoeman C, et al. Tofacitinib (CP-690,550) in combination with methotrexate in patients with active rheumatoid arthritis with an inadequate response to tumour necrosis factor inhibitors: a randomised phase 3 trial. *Lancet (London, England).* 2013;381(9865):451-460.
23. Burmester GR, Durez P, Shestakova G, et al. Association of HLA-DRB1 alleles with clinical responses to the anti-interleukin-17A monoclonal antibody secukinumab in active rheumatoid arthritis. *Rheumatology (Oxford, England).* 2016;55(1):49-55.
24. Burmester GR, Feist E, Sleeman MA, Wang B, White B, Magrini F. Mavrilimumab, a human monoclonal antibody targeting GM-CSF receptor-alpha, in subjects with rheumatoid arthritis: a randomised, double-blind, placebo-controlled, phase I, first-in-human study. *Annals of the rheumatic diseases.* 2011;70(9):1542-1549.
25. Burmester GR, Kivitz AJ, Kupper H, et al. Efficacy and safety of ascending methotrexate dose in combination with adalimumab: the randomised CONCERTO trial. *Annals of the rheumatic diseases.* 2015;74(6):1037-1044.
26. Burmester GR, Lin Y, Patel R, et al. Efficacy and safety of sarilumab monotherapy versus adalimumab monotherapy for the treatment of patients with active rheumatoid arthritis (MONARCH): a randomised, double-blind, parallel-group phase III trial. *Annals of the rheumatic diseases.* 2017;76(5):840-847.
27. Burmester GR, McInnes IB, Kremer J, et al. A randomised phase IIb study of mavrilimumab, a novel GM-CSF receptor alpha monoclonal antibody, in the treatment of rheumatoid arthritis. *Annals of the rheumatic diseases.* 2017;76(6):1020-1030.
28. Burmester GR, Rigby WF, van Vollenhoven RF, et al. Tocilizumab in early progressive rheumatoid arthritis: FUNCTION, a randomised controlled trial. *Annals of the rheumatic diseases.* 2016;75(6):1081-1091.
29. Burmester GR, Rubbert-Roth A, Cantagrel A, et al. A randomised, double-blind, parallel-group study of the safety and efficacy of subcutaneous tocilizumab versus intravenous tocilizumab in combination with traditional disease-modifying antirheumatic drugs in patients with moderate to severe rheumatoid arthritis (SUMMACTA study). *Annals of the rheumatic diseases.* 2014;73(1):69-74.
30. Burmester GR, Weinblatt ME, McInnes IB, et al. Efficacy and safety of mavrilimumab in subjects with rheumatoid arthritis. *Annals of the rheumatic diseases.* 2013;72(9):1445-1452.
31. Buttgereit F, Doering G, Schaeffler A, et al. Efficacy of modified-release versus standard prednisone to reduce duration of morning stiffness of the joints in rheumatoid arthritis (CAPRA-1): a double-blind, randomised controlled trial. *Lancet (London, England).* 2008;371(9608):205-214.
32. Buttgereit F, Mehta D, Kirwan J, et al. Low-dose prednisone chronotherapy for rheumatoid arthritis: a randomised clinical trial (CAPRA-2). *Annals of the rheumatic diseases.* 2013;72(2):204-210.
33. Cardiel MH, Tak PP, Bensen W, et al. A phase 2 randomized, double-blind study of AMG 108, a fully human monoclonal antibody to IL-1R, in patients with rheumatoid arthritis. *Arthritis research & therapy.* 2010;12(5):R192.
34. Chen DY, Chou SJ, Hsieh TY, et al. Randomized, double-blind, placebo-controlled, comparative study of human anti-TNF antibody adalimumab in combination with methotrexate and methotrexate alone in Taiwanese patients with active rheumatoid arthritis. *Journal of the Formosan Medical Association = Taiwan yi zhi.* 2009;108(4):310-319.
35. Chen XX, Dai Q, Huang AB, et al. A multicenter, randomized, double-blind clinical trial of combination therapy with Anbainuo, a novel recombinant human TNFRII:Fc fusion protein, plus methotrexate versus methotrexate alone or Anbainuo alone in Chinese patients with moderate to severe rheumatoid arthritis. *Clinical rheumatology.* 2013;32(1):99-108.
36. Chen XX, Li ZG, Wu HX, et al. A randomized, controlled trial of efficacy and safety of Anbainuo, a bio-similar etanercept, for moderate to severe rheumatoid arthritis inadequately responding to methotrexate. *Clinical rheumatology.* 2016;35(9):2175-2183.
37. Choe JY, Prodanovic N, Niebrzydowski J, et al. A randomised, double-blind, phase III study comparing SB2, an infliximab biosimilar, to the infliximab reference product Remicade in patients with moderate to severe rheumatoid arthritis despite methotrexate therapy. *Annals of the rheumatic diseases.* 2017;76(1):58-64.
38. Choy E, McKenna F, Vencovsky J, et al. Certolizumab pegol plus MTX administered every 4 weeks is effective in patients with RA who are partial responders to MTX. *Rheumatology (Oxford, England).* 2012;51(7):1226-1234.
39. Choy EH, Bendit M, McAleer D, et al. Safety, tolerability, pharmacokinetics and pharmacodynamics of an anti-oncostatin M monoclonal antibody in rheumatoid arthritis: results from phase II randomized, placebo-controlled trials. *Arthritis research & therapy.* 2013;15(5):R132.

© 2019 Strait A et al. *JAMA Network Open.*

40. Cohen S, Emery P, Greenwald M, et al. A phase I pharmacokinetics trial comparing PF-05280586 (a potential biosimilar) and rituximab in patients with active rheumatoid arthritis. *British journal of clinical pharmacology.* 2016;82(1):129-138.
41. Cohen S, Genovese MC, Choy E, et al. Efficacy and safety of the biosimilar ABP 501 compared with adalimumab in patients with moderate to severe rheumatoid arthritis: a randomised, double-blind, phase III equivalence study. *Annals of the rheumatic diseases.* 2017;76(10):1679-1687.
42. Cohen SB, Cheng TT, Chindalore V, et al. Evaluation of the efficacy and safety of pamapimod, a p38 MAP kinase inhibitor, in a double-blind, methotrexate-controlled study of patients with active rheumatoid arthritis. *Arthritis and rheumatism.* 2009;60(2):335-344.
43. Cohen SB, Dore RK, Lane NE, et al. Denosumab treatment effects on structural damage, bone mineral density, and bone turnover in rheumatoid arthritis: a twelve-month, multicenter, randomized, double-blind, placebo-controlled, phase II clinical trial. *Arthritis and rheumatism.* 2008;58(5):1299-1309.
44. Conaghan PG, Durez P, Alten RE, et al. Impact of intravenous abatacept on synovitis, osteitis and structural damage in patients with rheumatoid arthritis and an inadequate response to methotrexate: the ASSET randomised controlled trial. *Annals of the rheumatic diseases.* 2013;72(8):1287-1294.
45. Conaghan PG, Ostergaard M, Bowes MA, et al. Comparing the effects of tofacitinib, methotrexate and the combination, on bone marrow oedema, synovitis and bone erosion in methotrexate-naive, early active rheumatoid arthritis: results of an exploratory randomised MRI study incorporating semiquantitative and quantitative techniques. *Annals of the rheumatic diseases.* 2016;75(6):1024-1033.
46. Cutolo M, Bolosiu H, Perdriset G. Efficacy and safety of leflunomide in DMARD-naive patients with early rheumatoid arthritis: comparison of a loading and a fixed-dose regimen. *Rheumatology (Oxford, England).* 2013;52(6):1132-1140.
47. d'Elia HF, Carlsten H. The impact of hormone replacement therapy on humoral and cell-mediated immune responses in vivo in post-menopausal women with rheumatoid arthritis. *Scandinavian journal of immunology.* 2008;68(6):661-667.
48. Damjanov N, Tlustochowicz M, Aelion J, et al. Safety and Efficacy of SBI-087, a Subcutaneous Agent for B Cell Depletion, in Patients with Active Rheumatoid Arthritis: Results from a Phase II Randomized, Double-blind, Placebo-controlled Study. *The Journal of rheumatology.* 2016;43(12):2094-2100.
49. Deodhar A, Bitman B, Yang Y, Collier DH. The effect of etanercept on traditional metabolic risk factors for cardiovascular disease in patients with rheumatoid arthritis. *Clinical rheumatology.* 2016;35(12):3045-3052.
50. Detert J, Bastian H, Listing J, et al. Induction therapy with adalimumab plus methotrexate for 24 weeks followed by methotrexate monotherapy up to week 48 versus methotrexate therapy alone for DMARD-naive patients with early rheumatoid arthritis: HIT HARD, an investigator-initiated study. *Annals of the rheumatic diseases.* 2013;72(6):844-850.
51. Dougados M, Kissel K, Sheeran T, et al. Adding tocilizumab or switching to tocilizumab monotherapy in methotrexate inadequate responders: 24-week symptomatic and structural results of a 2-year randomised controlled strategy trial in rheumatoid arthritis (ACT-RAY). *Annals of the rheumatic diseases.* 2013;72(1):43-50.
52. Dougados M van der Heijde D, Chen YC, et al. Baricitinib in patients with inadequate response or intolerance to conventional synthetic DMARDs: results from the RA-BUILD study. *Annals of the rheumatic diseases.* 2017;76(1):88-95.
53. Durez P, Vandepapeliere P, Miranda P, et al. Therapeutic vaccination with TNF-Kinoid in TNF antagonist-resistant rheumatoid arthritis: a phase II randomized, controlled clinical trial. *PloS one.* 2014;9(12):e113465.
54. Edwards CJ, Ostor AJK, Naisbett-Groet B, Kiely P. Tapering versus steady-state methotrexate in combination with tocilizumab for rheumatoid arthritis: a randomized, double-blind trial. *Rheumatology (Oxford, England).* 2018;57(1):84-91.
55. Emery P, Bingham CO, 3rd, Burmester GR, et al. Certolizumab pegol in combination with dose-optimised methotrexate in DMARD-naive patients with early, active rheumatoid arthritis with poor prognostic factors: 1-year results from C-EARLY, a randomised, double-blind, placebo-controlled phase III study. *Annals of the rheumatic diseases.* 2017;76(1):96-104.
56. Emery P, Breedveld FC, Hall S, et al. Comparison of methotrexate monotherapy with a combination of methotrexate and etanercept in active, early, moderate to severe rheumatoid arthritis (COMET): a randomised, double-blind, parallel treatment trial. *Lancet (London, England).* 2008;372(9636):375-382.
57. Emery P, Burmester GR, Bykerk VP, et al. Evaluating drug-free remission with abatacept in early rheumatoid arthritis: results from the phase 3b, multicentre, randomised, active-controlled AVERT study of 24 months, with a 12-month, double-blind treatment period. *Annals of the rheumatic diseases.* 2015;74(1):19-26.

58. Emery P, Deodhar A, Rigby WF, et al. Efficacy and safety of different doses and retreatment of rituximab: a randomised, placebo-controlled trial in patients who are biological naive with active rheumatoid arthritis and an inadequate response to methotrexate (Study Evaluating Rituximab's Efficacy in MTX iNadequate rEsponders (SERENE)). *Annals of the rheumatic diseases.* 2010;69(9):1629-1635.
59. Emery P, Fleischmann RM, Moreland LW, et al. Golimumab, a human anti-tumor necrosis factor alpha monoclonal antibody, injected subcutaneously every four weeks in methotrexate-naive patients with active rheumatoid arthritis: twenty-four-week results of a phase III, multicenter, randomized, double-blind, placebo-controlled study of golimumab before methotrexate as first-line therapy for early-onset rheumatoid arthritis. *Arthritis and rheumatism.* 2009;60(8):2272-2283.
60. Emery P, Hammoudeh M, FitzGerald O, et al. Sustained remission with etanercept tapering in early rheumatoid arthritis. *The New England journal of medicine.* 2014;371(19):1781-1792.
61. Emery P, Keystone E, Tony HP, et al. IL-6 receptor inhibition with tocilizumab improves treatment outcomes in patients with rheumatoid arthritis refractory to anti-tumour necrosis factor biologicals: results from a 24-week multicentre randomised placebo-controlled trial. *Annals of the rheumatic diseases.* 2008;67(11):1516-1523.
62. Emery P, Vencovsky J, Sylwestrzak A, et al. A phase III randomised, double-blind, parallel-group study comparing SB4 with etanercept reference product in patients with active rheumatoid arthritis despite methotrexate therapy. *Annals of the rheumatic diseases.* 2017;76(1):51-57.
63. Emu B, Luca D, Offutt C, et al. Safety, pharmacokinetics, and biologic activity of pateclizumab, a novel monoclonal antibody targeting lymphotoxin alpha: results of a phase I randomized, placebo-controlled trial. *Arthritis research & therapy.* 2012;14(1):R6.
64. Fan W, Zhao DB, Hu SX, et al. A randomized, double-blind, and placebo-controlled multicenter clinical trial of a novel cytotoxic T-lymphocyte antigen-4 fusion protein, Leining, in Chinese active rheumatoid arthritis patients with an inadequate response to methotrexate. *Rheumatology international.* 2014;34(11):1519-1527.
65. Fleischmann R, Cutolo M, Genovese MC, et al. Phase IIb dose-ranging study of the oral JAK inhibitor tofacitinib (CP-690,550) or adalimumab monotherapy versus placebo in patients with active rheumatoid arthritis with an inadequate response to disease-modifying antirheumatic drugs. *Arthritis and rheumatism.* 2012;64(3):617-629.
66. Fleischmann R, Kremer J, Cush J, et al. Placebo-controlled trial of tofacitinib monotherapy in rheumatoid arthritis. *The New England journal of medicine.* 2012;367(6):495-507.
67. Fleischmann R, Mysler E, Hall S, et al. Efficacy and safety of tofacitinib monotherapy, tofacitinib with methotrexate, and adalimumab with methotrexate in patients with rheumatoid arthritis (ORAL Strategy): a phase 3b/4, double-blind, head-to-head, randomised controlled trial. *Lancet (London, England).* 2017;390(10093):457-468.
68. Fleischmann R, Schiff M, van der Heijde D, et al. Baricitinib, Methotrexate, or Combination in Patients with Rheumatoid Arthritis and No or Limited Prior Disease-Modifying Antirheumatic Drug Treatment. *Arthritis & rheumatology (Hoboken, NJ).* 2017;69(3):506-517.
69. Fleischmann R, van Adelsberg J, Lin Y, et al. Sarilumab and Nonbiologic Disease-Modifying Antirheumatic Drugs in Patients with Active Rheumatoid Arthritis and Inadequate Response or Intolerance to Tumor Necrosis Factor Inhibitors. *Arthritis & rheumatology (Hoboken, NJ).* 2017;69(2):277-290.
70. Fleischmann R, Vencovsky J, van Vollenhoven RF, et al. Efficacy and safety of certolizumab pegol monotherapy every 4 weeks in patients with rheumatoid arthritis failing previous disease-modifying antirheumatic therapy: the FAST4WARD study. *Annals of the rheumatic diseases.* 2009;68(6):805-811.
71. Fleischmann RM, Damjanov NS, Kivitz AJ, Legedza A, Hoock T, Kinnman N. A randomized, double-blind, placebo-controlled, twelve-week, dose-ranging study of decernotinib, an oral selective JAK-3 inhibitor, as monotherapy in patients with active rheumatoid arthritis. *Arthritis & rheumatology (Hoboken, NJ).* 2015;67(2):334-343.
72. Fleishaker DL, Garcia Meijide JA, Petrov A, et al. Maraviroc, a chemokine receptor-5 antagonist, fails to demonstrate efficacy in the treatment of patients with rheumatoid arthritis in a randomized, double-blind placebo-controlled trial. *Arthritis research & therapy.* 2012;14(1):R11.
73. Furst DE, Shaikh SA, Greenwald M, et al. Two dosing regimens of certolizumab pegol in patients with active rheumatoid arthritis. *Arthritis care & research.* 2015;67(2):151-160.
74. Gabay C, Emery P, van Vollenhoven R, et al. Tocilizumab monotherapy versus adalimumab monotherapy for treatment of rheumatoid arthritis (ADACTA): a randomised, double-blind, controlled phase 4 trial. *Lancet (London, England).* 2013;381(9877):1541-1550.
75. Genovese MC, Bojin S, Biagini IM, et al. Tabalumab in rheumatoid arthritis patients with an inadequate response to methotrexate and naive to biologic therapy: a phase II, randomized, placebo-controlled trial. *Arthritis and rheumatism.* 2013;65(4):880-889.

© 2019 Strait A et al. *JAMA Network Open.*

76. Genovese MC, Cohen SB, Wofsy D, et al. A 24-week, randomized, double-blind, placebo-controlled, parallel group study of the efficacy of oral SCIO-469, a p38 mitogen-activated protein kinase inhibitor, in patients with active rheumatoid arthritis. *The Journal of rheumatology.* 2011;38(5):846-854.
77. Genovese MC, Covarrubias A, Leon G, et al. Subcutaneous abatacept versus intravenous abatacept: a phase IIIb noninferiority study in patients with an inadequate response to methotrexate. *Arthritis and rheumatism.* 2011;63(10):2854-2864.
78. Genovese MC, Durez P, Richards HB, et al. Efficacy and safety of secukinumab in patients with rheumatoid arthritis: a phase II, dose-finding, double-blind, randomised, placebo-controlled study. *Annals of the rheumatic diseases.* 2013;72(6):863-869.
79. Genovese MC, Fleischmann R, Furst D, et al. Efficacy and safety of olokizumab in patients with rheumatoid arthritis with an inadequate response to TNF inhibitor therapy: outcomes of a randomised Phase IIb study. *Annals of the rheumatic diseases.* 2014;73(9):1607-1615.
80. Genovese MC, Fleischmann R, Kivitz AJ, et al. Sarilumab Plus Methotrexate in Patients with Active Rheumatoid Arthritis and Inadequate Response to Methotrexate: Results of a Phase III Study. *Arthritis & rheumatology (Hoboken, NJ).* 2015;67(6):1424-1437.
81. Genovese MC, Fleischmann RM, Greenwald M, et al. Tabalumab, an anti-BAFF monoclonal antibody, in patients with active rheumatoid arthritis with an inadequate response to TNF inhibitors. *Annals of the rheumatic diseases.* 2013;72(9):1461-1468.
82. Genovese MC, Greenwald M, Cho CS, et al. A phase II randomized study of subcutaneous ixekizumab, an anti-interleukin-17 monoclonal antibody, in rheumatoid arthritis patients who were naive to biologic agents or had an inadequate response to tumor necrosis factor inhibitors. *Arthritis & rheumatology (Hoboken, NJ).* 2014;66(7):1693-1704.
83. Genovese MC, Greenwald M, Codding C, et al. Peficitinib, a JAK Inhibitor, in Combination with Limited Conventional Synthetic Disease-Modifying Antirheumatic Drugs in the Treatment of Moderate-to-Severe Rheumatoid Arthritis. *Arthritis & rheumatology (Hoboken, NJ).* 2017;69(5):932-942.
84. Genovese MC, Hsia E, Belkowski SM, et al. Results from a Phase IIA Parallel Group Study of JNJ-40346527, an Oral CSF-1R Inhibitor, in Patients with Active Rheumatoid Arthritis despite Disease-modifying Antirheumatic Drug Therapy. *The Journal of rheumatology.* 2015;42(10):1752-1760.
85. Genovese MC, Jarosova K, Cieslak D, et al. Apremilast in Patients with Active Rheumatoid Arthritis: A Phase II, Multicenter, Randomized, Double-Blind, Placebo-Controlled, Parallel-Group Study. *Arthritis & rheumatology (Hoboken, NJ).* 2015;67(7):1703-1710.
86. Genovese MC, Kaine JL, Lowenstein MB, et al. Ocrelizumab, a humanized anti-CD20 monoclonal antibody, in the treatment of patients with rheumatoid arthritis: a phase I/II randomized, blinded, placebo-controlled, dose-ranging study. *Arthritis and rheumatism.* 2008;58(9):2652-2661.
87. Genovese MC, Kavanaugh A, Weinblatt ME, et al. An oral Syk kinase inhibitor in the treatment of rheumatoid arthritis: a three-month randomized, placebo-controlled, phase II study in patients with active rheumatoid arthritis that did not respond to biologic agents. *Arthritis and rheumatism.* 2011;63(2):337-345.
88. Genovese MC, Kinnman N, de La Bourdonnaye G, Pena Rossi C, Tak PP. Atacicept in patients with rheumatoid arthritis and an inadequate response to tumor necrosis factor antagonist therapy: results of a phase II, randomized, placebo-controlled, dose-finding trial. *Arthritis and rheumatism.* 2011;63(7):1793-1803.
89. Genovese MC, Kremer J, Zamani O, et al. Baricitinib in Patients with Refractory Rheumatoid Arthritis. *The New England journal of medicine.* 2016;374(13):1243-1252.
90. Genovese MC, Lee E, Satterwhite J, et al. A phase 2 dose-ranging study of subcutaneous tabalumab for the treatment of patients with active rheumatoid arthritis and an inadequate response to methotrexate. *Annals of the rheumatic diseases.* 2013;72(9):1453-1460.
91. Genovese MC, McKay JD, Nasonov EL, et al. Interleukin-6 receptor inhibition with tocilizumab reduces disease activity in rheumatoid arthritis with inadequate response to disease-modifying antirheumatic drugs: the tocilizumab in combination with traditional disease-modifying antirheumatic drug therapy study. *Arthritis and rheumatism.* 2008;58(10):2968-2980.
92. Genovese MC, Silverman GJ, Emery P, et al. Efficacy and Safety of Tabalumab, an Anti-B-Cell-Activating Factor Monoclonal Antibody, in a Heterogeneous Rheumatoid Arthritis Population: Results from a Randomized, Placebo-Controlled, Phase 3 Trial (FLEX-O). *Journal of clinical rheumatology : practical reports on rheumatic & musculoskeletal diseases.* 2015;21(5):231-238.
93. Genovese MC, Smolen JS, Weinblatt ME, et al. Efficacy and Safety of ABT-494, a Selective JAK-1 Inhibitor, in a Phase IIb Study in Patients with Rheumatoid Arthritis and an Inadequate Response to Methotrexate. *Arthritis & rheumatology (Hoboken, NJ).* 2016;68(12):2857-2866.

© 2019 Strait A et al. *JAMA Network Open.*

94. Genovese MC, Van den Bosch F, Roberson SA, et al. LY2439821, a humanized anti-interleukin-17 monoclonal antibody, in the treatment of patients with rheumatoid arthritis: A phase I randomized, double-blind, placebo-controlled, proof-of-concept study. *Arthritis and rheumatism.* 2010;62(4):929-939.
95. Genovese MC, van der Heijde DM, Keystone EC, et al. A phase III, multicenter, randomized, double-blind, placebo-controlled, parallel-group study of 2 dosing regimens of fostamatinib in patients with rheumatoid arthritis with an inadequate response to a tumor necrosis factor-alpha antagonist. *The Journal of rheumatology.* 2014;41(11):2120-2128.
96. Genovese MC, van Vollenhoven RF, Pacheco-Tena C, Zhang Y, Kinnman N. VX-509 (Decernotinib), an Oral Selective JAK-3 Inhibitor, in Combination with Methotrexate in Patients with Rheumatoid Arthritis. *Arthritis & rheumatology (Hoboken, NJ).* 2016;68(1):46-55.
97. Genovese MC, Yang F, Ostergaard M, Kinnman N. Efficacy of VX-509 (decernotinib) in combination with a disease-modifying antirheumatic drug in patients with rheumatoid arthritis: clinical and MRI findings. *Annals of the rheumatic diseases.* 2016;75(11):1979-1983.
98. Greenwald MW, Shergy WJ, Kaine JL, Sweetser MT, Gilder K, Linnik MD. Evaluation of the safety of rituximab in combination with a tumor necrosis factor inhibitor and methotrexate in patients with active rheumatoid arthritis: results from a randomized controlled trial. *Arthritis and rheumatism.* 2011;63(3):622-632.
99. Gupta P, Friberg LE, Karlsson MO, Krishnaswami S, French J. A semi-mechanistic model of CP-690,550-induced reduction in neutrophil counts in patients with rheumatoid arthritis. *Journal of clinical pharmacology.* 2010;50(6):679-687.
100. Harigai M, Tanaka Y, Maisawa S. Safety and efficacy of various dosages of ocrelizumab in Japanese patients with rheumatoid arthritis with an inadequate response to methotrexate therapy: a placebo-controlled double-blind parallel-group study. *The Journal of rheumatology.* 2012;39(3):486-495.
101. He Y, Lu A, Lu C, et al. Symptom combinations assessed in traditional Chinese medicine and its predictive role in ACR20 efficacy response in rheumatoid arthritis. *The American journal of Chinese medicine.* 2008;36(4):675-683.
102. Hobl EL, Mader RM, Erlacher L, et al. The influence of methotrexate on the gene expression of the pro-inflammatory cytokine IL-12A in the therapy of rheumatoid arthritis. *Clinical and experimental rheumatology.* 2011;29(6):963-969.
103. Hobl EL, Mader RM, Jilma B, et al. A randomized, double-blind, parallel, single-site pilot trial to compare two different starting doses of methotrexate in methotrexate-naive adult patients with rheumatoid arthritis. *Clinical therapeutics.* 2012;34(5):1195-1203.
104. Horslev-Petersen K, Hetland ML, Junker P, et al. Adalimumab added to a treat-to-target strategy with methotrexate and intra-articular triamcinolone in early rheumatoid arthritis increased remission rates, function and quality of life. The OPERA Study: an investigator-initiated, randomised, double-blind, parallel-group, placebo-controlled trial. *Annals of the rheumatic diseases.* 2014;73(4):654-661.
105. Hu D, Bao C, Chen S, et al. A comparison study of a recombinant tumor necrosis factor receptor:Fc fusion protein (rhTNFR:Fc) and methotrexate in treatment of patients with active rheumatoid arthritis in China. *Rheumatology international.* 2009;29(3):297-303.
106. Huffstutter JE, Kafka S, Brent LH, et al. Clinical response to golimumab in rheumatoid arthritis patients who were receiving etanercept or adalimumab: results of a multicenter active treatment study. *Current medical research and opinion.* 2017;33(4):657-666.
107. Huizinga TW, Batalov A, Stoilov R, et al. Phase 1b randomized, double-blind study of namilumab, an anti-granulocyte macrophage colony-stimulating factor monoclonal antibody, in mild-to-moderate rheumatoid arthritis. *Arthritis research & therapy.* 2017;19(1):53.
108. Huizinga TW, Fleischmann RM, Jasson M, et al. Sarilumab, a fully human monoclonal antibody against IL-6Ralpha in patients with rheumatoid arthritis and an inadequate response to methotrexate: efficacy and safety results from the randomised SARIL-RA-MOBILITY Part A trial. *Annals of the rheumatic diseases.* 2014;73(9):1626-1634.
109. Ignatenko S, Skrumsager BK, Mouritzen U. Safety, PK, and PD of recombinant anti-interleukin-21 monoclonal antibody in a first-in-human trial. *International journal of clinical pharmacology and therapeutics.* 2016;54(4):243-252.
110. Ikonomidis I, Lekakis JP, Nikolaou M, et al. Inhibition of interleukin-1 by anakinra improves vascular and left ventricular function in patients with rheumatoid arthritis. *Circulation.* 2008;117(20):2662-2669.
111. Ikonomidis I, Tzortzis S, Andreadou I, et al. Increased benefit of interleukin-1 inhibition on vascular function, myocardial deformation, and twisting in patients with coronary artery disease and coexisting rheumatoid arthritis. *Circulation Cardiovascular imaging.* 2014;7(4):619-628.
112. Ishaq M, Muhammad JS, Hameed K, Mirza AI. Leflunomide or methotrexate? Comparison of clinical efficacy and safety in low socio-economic rheumatoid arthritis patients. *Modern rheumatology.* 2011;21(4):375-380.

113. Ishiguro N, Yamamoto K, Katayama K, et al. Concomitant iguratimod therapy in patients with active rheumatoid arthritis despite stable doses of methotrexate: a randomized, double-blind, placebo-controlled trial. *Modern rheumatology.* 2013;23(3):430-439.
114. Iwahashi M, Inoue H, Matsubara T, et al. Efficacy, safety, pharmacokinetics and immunogenicity of abatacept administered subcutaneously or intravenously in Japanese patients with rheumatoid arthritis and inadequate response to methotrexate: A Phase II/III, randomized study. *Modern rheumatology.* 2014;24(6):885-891.
115. Jaimes-Hernandez J, Melendez-Mercado CI, Mendoza-Fuentes A, Aranda-Pereira P, Castaneda-Hernandez G. Efficacy of leflunomide 100mg weekly compared to low dose methotrexate in patients with active rheumatoid arthritis. Double blind randomized clinical trial. *Reumatologia clinica.* 2012;8(5):243-249.
116. Jamshidi A, Gharibdoost F, Vojdanian M, et al. A phase III, randomized, two-armed, double-blind, parallel, active controlled, and non-inferiority clinical trial to compare efficacy and safety of biosimilar adalimumab (CinnoRA(R)) to the reference product (Humira(R)) in patients with active rheumatoid arthritis. *Arthritis research & therapy.* 2017;19(1):168.
117. Jani RH, Gupta R, Bhatia G, et al. A prospective, randomized, double-blind, multicentre, parallel-group, active controlled study to compare efficacy and safety of biosimilar adalimumab (Exemptia; ZRC-3197) and adalimumab (Humira) in patients with rheumatoid arthritis. *International journal of rheumatic diseases.* 2016;19(11):1157-1168.
118. Jones G, Sebba A, Gu J, et al. Comparison of tocilizumab monotherapy versus methotrexate monotherapy in patients with moderate to severe rheumatoid arthritis: the AMBITION study. *Annals of the rheumatic diseases.* 2010;69(1):88-96.
119. Kaeley GS, Evangelisto AM, Nishio MJ, et al. Methotrexate Dosage Reduction Upon Adalimumab Initiation: Clinical and Ultrasonographic Outcomes from the Randomized Noninferiority MUSICA Trial. *The Journal of rheumatology.* 2016;43(8):1480-1489.
120. Kass AS, Forre OT, Fagerland MW, Gulseth HC, Torjesen PA, Hollan I. Short-term treatment with a gonadotropin-releasing hormone antagonist, cetrorelix, in rheumatoid arthritis (AGRA): a randomized, double-blind, placebo-controlled study. *Scandinavian journal of rheumatology.* 2014;43(1):22-27.
121. Kavanaugh A, Fleischmann RM, Emery P, et al. Clinical, functional and radiographic consequences of achieving stable low disease activity and remission with adalimumab plus methotrexate or methotrexate alone in early rheumatoid arthritis: 26-week results from the randomised, controlled OPTIMA study. *Annals of the rheumatic diseases.* 2013;72(1):64-71.
122. Kavanaugh A, Kremer J, Ponce L, et al. Filgotinib (GLPG0634/GS-6034), an oral selective JAK1 inhibitor, is effective as monotherapy in patients with active rheumatoid arthritis: results from a randomised, dose-finding study (DARWIN 2). *Annals of the rheumatic diseases.* 2017;76(6):1009-1019.
123. Kawai S, Takeuchi T, Yamamoto K, Tanaka Y, Miyasaka N. Efficacy and safety of additional use of tacrolimus in patients with early rheumatoid arthritis with inadequate response to DMARDs--a multicenter, double-blind, parallel-group trial. *Modern rheumatology.* 2011;21(5):458-468.
124. Kay J, Matteson EL, Dasgupta B, et al. Golimumab in patients with active rheumatoid arthritis despite treatment with methotrexate: a randomized, double-blind, placebo-controlled, dose-ranging study. *Arthritis and rheumatism.* 2008;58(4):964-975.
125. Kennedy WP, Simon JA, Offutt C, et al. Efficacy and safety of pateclizumab (anti-lymphotoxin-alpha) compared to adalimumab in rheumatoid arthritis: a head-to-head phase 2 randomized controlled study (The ALTARA Study). *Arthritis research & therapy.* 2014;16(5):467.
126. Keystone E, Heijde D, Mason D, Jr., et al. Certolizumab pegol plus methotrexate is significantly more effective than placebo plus methotrexate in active rheumatoid arthritis: findings of a fifty-two-week, phase III, multicenter, randomized, double-blind, placebo-controlled, parallel-group study. *Arthritis and rheumatism.* 2008;58(11):3319-3329.
127. Keystone EC, Genovese MC, Klareskog L, et al. Golimumab, a human antibody to tumour necrosis factor {alpha} given by monthly subcutaneous injections, in active rheumatoid arthritis despite methotrexate therapy: the GO-FORWARD Study. *Annals of the rheumatic diseases.* 2009;68(6):789-796.
128. Keystone EC, Shirinsky VS, Simon LS, Pedder S, Hewitt LA. Efficacy and safety of CH-1504, a metabolically stable antifolate, in patients with active rheumatoid arthritis: results of a phase II multicenter randomized study. *The Journal of rheumatology.* 2011;38(9):1875-1883.
129. Keystone EC, Taylor PC, Drescher E, et al. Safety and efficacy of baricitinib at 24 weeks in patients with rheumatoid arthritis who have had an inadequate response to methotrexate. *Annals of the rheumatic diseases.* 2015;74(2):333-340.
130. Keystone EC, Wang MM, Layton M, Hollis S, McInnes IB. Clinical evaluation of the efficacy of the P2X7 purinergic receptor antagonist AZD9056 on the signs and symptoms of rheumatoid arthritis in patients with active

disease despite treatment with methotrexate or sulphasalazine. *Annals of the rheumatic diseases.* 2012;71(10):1630-1635.

131. Kim J, Ryu H, Yoo DH, et al. A clinical trial and extension study of infliximab in Korean patients with active rheumatoid arthritis despite methotrexate treatment. *Journal of Korean medical science.* 2013;28(12):1716-1722.

132. Kitas GD, Abreu G, Jedrychowicz-Rosiak K, et al. The effects of the spleen tyrosine kinase inhibitor fostamatinib on ambulatory blood pressure in patients with active rheumatoid arthritis: results of the OSKIRA-ABPM (ambulatory blood pressure monitoring) randomized trial. *Journal of the American Society of Hypertension : JASH.* 2014;8(11):780-790.

133. Kivitz A, Olech E, Borofsky M, et al. Subcutaneous tocilizumab versus placebo in combination with disease-modifying antirheumatic drugs in patients with rheumatoid arthritis. *Arthritis care & research.* 2014;66(11):1653-1661.

134. Kivitz AJ, Gutierrez-Urena SR, Poiley J, et al. Peficitinib, a JAK Inhibitor, in the Treatment of Moderate-to-Severe Rheumatoid Arthritis in Patients with an Inadequate Response to Methotrexate. *Arthritis & rheumatology (Hoboken, NJ).* 2017;69(4):709-719.

135. Koffeman EC, Genovese M, Amox D, et al. Epitope-specific immunotherapy of rheumatoid arthritis: clinical responsiveness occurs with immune deviation and relies on the expression of a cluster of molecules associated with T cell tolerance in a double-blind, placebo-controlled, pilot phase II trial. *Arthritis and rheumatism.* 2009;60(11):3207-3216.

136. Krausz S, Boumans MJ, Gerlag DM, et al. Brief report: a phase IIa, randomized, double-blind, placebo-controlled trial of apilimod mesylate, an interleukin-12/interleukin-23 inhibitor, in patients with rheumatoid arthritis. *Arthritis and rheumatism.* 2012;64(6):1750-1755.

137. Kremer J, Li ZG, Hall S, et al. Tofacitinib in combination with nonbiologic disease-modifying antirheumatic drugs in patients with active rheumatoid arthritis: a randomized trial. *Annals of internal medicine.* 2013;159(4):253-261.

138. Kremer J, Ritchlin C, Mendelsohn A, et al. Golimumab, a new human anti-tumor necrosis factor alpha antibody, administered intravenously in patients with active rheumatoid arthritis: Forty-eight-week efficacy and safety results of a phase III randomized, double-blind, placebo-controlled study. *Arthritis and rheumatism.* 2010;62(4):917-928.

139. Kremer JM, Blanco R, Brzosko M, et al. Tocilizumab inhibits structural joint damage in rheumatoid arthritis patients with inadequate responses to methotrexate: results from the double-blind treatment phase of a randomized placebo-controlled trial of tocilizumab safety and prevention of structural joint damage at one year. *Arthritis and rheumatism.* 2011;63(3):609-621.

140. Kremer JM, Bloom BJ, Breedveld FC, et al. The safety and efficacy of a JAK inhibitor in patients with active rheumatoid arthritis: Results of a double-blind, placebo-controlled phase IIa trial of three dosage levels of CP-690,550 versus placebo. *Arthritis and rheumatism.* 2009;60(7):1895-1905.

141. Kremer JM, Cohen S, Wilkinson BE, et al. A phase IIb dose-ranging study of the oral JAK inhibitor tofacitinib (CP-690,550) versus placebo in combination with background methotrexate in patients with active rheumatoid arthritis and an inadequate response to methotrexate alone. *Arthritis and rheumatism.* 2012;64(4):970-981.

142. Kremer JM, Emery P, Camp HS, et al. A Phase IIb Study of ABT-494, a Selective JAK-1 Inhibitor, in Patients with Rheumatoid Arthritis and an Inadequate Response to Anti-Tumor Necrosis Factor Therapy. *Arthritis & rheumatology (Hoboken, NJ).* 2016;68(12):2867-2877.

143. Kremer JM, Kivitz AJ, Simon-Campos JA, et al. Evaluation of the effect of tofacitinib on measured glomerular filtration rate in patients with active rheumatoid arthritis: results from a randomised controlled trial. *Arthritis research & therapy.* 2015;17:95.

144. Landewe RB, Houbiers JG, Van den Bosch F, et al. Intranasal administration of recombinant human cartilage glycoprotein-39 as a treatment for rheumatoid arthritis: a phase II, multicentre, double-blind, randomised, placebo-controlled, parallel-group, dose-finding trial. *Annals of the rheumatic diseases.* 2010;69(9):1655-1659.

145. Lee EB, Fleischmann R, Hall S, et al. Tofacitinib versus methotrexate in rheumatoid arthritis. *The New England journal of medicine.* 2014;370(25):2377-2386.

146. Leirisalo-Repo M, Kautiainen H, Laasonen L, et al. Infliximab for 6 months added on combination therapy in early rheumatoid arthritis: 2-year results from an investigator-initiated, randomised, double-blind, placebo-controlled study (the NEO-RACo Study). *Annals of the rheumatic diseases.* 2013;72(6):851-857.

147. Li Z, Zhang F, Kay J, et al. Efficacy and safety results from a Phase 3, randomized, placebo-controlled trial of subcutaneous golimumab in Chinese patients with active rheumatoid arthritis despite methotrexate therapy. *International journal of rheumatic diseases.* 2016;19(11):1143-1156.

148. Lu LJ, Bao CD, Dai M, et al. Multicenter, randomized, double-blind, controlled trial of treatment of active rheumatoid arthritis with T-614 compared with methotrexate. *Arthritis and rheumatism.* 2009;61(7):979-987.

© 2019 Strait A et al. *JAMA Network Open.*

149. Lu LJ, Teng JL, Bao CD, et al. Safety and efficacy of T-614 in the treatment of patients with active rheumatoid arthritis: a double blind, randomized, placebo-controlled and multicenter trial. *Chinese medical journal.* 2008;121(7):615-619.
150. Marder W, Khalatbari S, Myles JD, et al. The peroxisome proliferator activated receptor-gamma pioglitazone improves vascular function and decreases disease activity in patients with rheumatoid arthritis. *Journal of the American Heart Association.* 2013;2(6):e000441.
151. Martin DA, Churchill M, Flores-Suarez L, et al. A phase Ib multiple ascending dose study evaluating safety, pharmacokinetics, and early clinical response of brodalumab, a human anti-IL-17R antibody, in methotrexate-resistant rheumatoid arthritis. *Arthritis research & therapy.* 2013;15(5):R164.
152. McInnes IB, Thompson L, Giles JT, et al. Effect of interleukin-6 receptor blockade on surrogates of vascular risk in rheumatoid arthritis: MEASURE, a randomised, placebo-controlled study. *Annals of the rheumatic diseases.* 2015;74(4):694-702.
153. Mease P, Strand V, Shalamberidze L, et al. A phase II, double-blind, randomised, placebo-controlled study of BMS945429 (ALD518) in patients with rheumatoid arthritis with an inadequate response to methotrexate. *Annals of the rheumatic diseases.* 2012;71(7):1183-1189.
154. Mease PJ, Cohen S, Gaylis NB, et al. Efficacy and safety of retreatment in patients with rheumatoid arthritis with previous inadequate response to tumor necrosis factor inhibitors: results from the SUNRISE trial. *The Journal of rheumatology.* 2010;37(5):917-927.
155. Miyasaka N. Clinical investigation in highly disease-affected rheumatoid arthritis patients in Japan with adalimumab applying standard and general evaluation: the CHANGE study. *Modern rheumatology.* 2008;18(3):252-262.
156. Moreland LW, O'Dell JR, Paulus HE, et al. A randomized comparative effectiveness study of oral triple therapy versus etanercept plus methotrexate in early aggressive rheumatoid arthritis: the treatment of Early Aggressive Rheumatoid Arthritis Trial. *Arthritis and rheumatism.* 2012;64(9):2824-2835.
157. Nam JL, Villeneuve E, Hensor EM, et al. Remission induction comparing infliximab and high-dose intravenous steroid, followed by treat-to-target: a double-blind, randomised, controlled trial in new-onset, treatment-naive, rheumatoid arthritis (the IDEA study). *Annals of the rheumatic diseases.* 2014;73(1):75-85.
158. Nishimoto N, Miyasaka N, Yamamoto K, et al. Study of active controlled tocilizumab monotherapy for rheumatoid arthritis patients with an inadequate response to methotrexate (SATORI): significant reduction in disease activity and serum vascular endothelial growth factor by IL-6 receptor inhibition therapy. *Modern rheumatology.* 2009;19(1):12-19.
159. O'Dell JR, Mikuls TR, Taylor TH, et al. Therapies for active rheumatoid arthritis after methotrexate failure. *The New England journal of medicine.* 2013;369(4):307-318.
160. O'Neill F, Charakida M, Topham E, et al. Anti-inflammatory treatment improves high-density lipoprotein function in rheumatoid arthritis. *Heart (British Cardiac Society).* 2017;103(10):766-773.
161. Ogata A, Tanimura K, Sugimoto T, et al. Phase III study of the efficacy and safety of subcutaneous versus intravenous tocilizumab monotherapy in patients with rheumatoid arthritis. *Arthritis care & research.* 2014;66(3):344-354.
162. Ogrendik M. Efficacy of roxithromycin in adult patients with rheumatoid arthritis who had not received disease-modifying antirheumatic drugs: a 3-month, randomized, double-blind, placebo-controlled trial. *Clinical therapeutics.* 2009;31(8):1754-1764.
163. Ogrendik M, Karagoz N. Treatment of rheumatoid arthritis with roxithromycin: a randomized trial. *Postgraduate medicine.* 2011;123(5):220-227.
164. Ormseth MJ, Oeser AM, Cunningham A, et al. Peroxisome proliferator-activated receptor gamma agonist effect on rheumatoid arthritis: a randomized controlled trial. *Arthritis research & therapy.* 2013;15(5):R110.
165. Ostergaard M, Baslund B, Rigby W, et al. Ofatumumab, a human anti-CD20 monoclonal antibody, for treatment of rheumatoid arthritis with an inadequate response to one or more disease-modifying antirheumatic drugs: results of a randomized, double-blind, placebo-controlled, phase I/II study. *Arthritis and rheumatism.* 2010;62(8):2227-2238.
166. Ostergaard M, Jacobsson LT, Schaufelberger C, et al. MRI assessment of early response to certolizumab pegol in rheumatoid arthritis: a randomised, double-blind, placebo-controlled phase IIIb study applying MRI at weeks 0, 1, 2, 4, 8 and 16. *Annals of the rheumatic diseases.* 2015;74(6):1156-1163.
167. Pavelka K, Chon Y, Newmark R, Lin SL, Baumgartner S, Erondu N. A study to evaluate the safety, tolerability, and efficacy of brodalumab in subjects with rheumatoid arthritis and an inadequate response to methotrexate. *The Journal of rheumatology.* 2015;42(6):912-919.
168. Pavelka K, Jarosova K, Suchy D, et al. Increasing the infliximab dose in rheumatoid arthritis patients: a randomised, double blind study failed to confirm its efficacy. *Annals of the rheumatic diseases.* 2009;68(8):1285-1289.

© 2019 Strait A et al. *JAMA Network Open.*

169. Peterfy C, Emery P, Tak PP, et al. MRI assessment of suppression of structural damage in patients with rheumatoid arthritis receiving rituximab: results from the randomised, placebo-controlled, double-blind RA-SCORE study. *Annals of the rheumatic diseases.* 2016;75(1):170-177.
170. Petrov VI, Babaeva AR, Solodenkova KS, et al. Effect of combination of anticytokine preparations anaferon and artrofoon on immune inflammation in rheumatoid arthritis. *Bulletin of experimental biology and medicine.* 2009;148(3):489-492.
171. Pincus T, Swearingen CJ, Luta G, Sokka T. Efficacy of prednisone 1-4 mg/day in patients with rheumatoid arthritis: a randomised, double-blind, placebo-controlled withdrawal clinical trial. *Annals of the rheumatic diseases.* 2009;68(11):1715-1720.
172. Rigby W, Tony HP, Oelke K, et al. Safety and efficacy of ocrelizumab in patients with rheumatoid arthritis and an inadequate response to methotrexate: results of a forty-eight-week randomized, double-blind, placebo-controlled, parallel-group phase III trial. *Arthritis and rheumatism.* 2012;64(2):350-359.
173. Roman-Blas JA, Castaneda S, Cutolo M, Herrero-Beaumont G. Efficacy and safety of a selective estrogen receptor beta agonist, ERB-041, in patients with rheumatoid arthritis: a 12-week, randomized, placebo-controlled, phase II study. *Arthritis care & research.* 2010;62(11):1588-1593.
174. Roux CH, Breuil V, Valerio L, et al. Etanercept compared to intraarticular corticosteroid injection in rheumatoid arthritis: double-blind, randomized pilot study. *The Journal of rheumatology.* 2011;38(6):1009-1011.
175. Rubbert-Roth A, Tak PP, Zerbini C, et al. Efficacy and safety of various repeat treatment dosing regimens of rituximab in patients with active rheumatoid arthritis: results of a Phase III randomized study (MIRROR). *Rheumatology (Oxford, England).* 2010;49(9):1683-1693.
176. Sadra V, Khabbazi A, Kolahi S, Hajialiloo M, Ghojazadeh M. Randomized double-blind study of the effect of dexamethasone and methylprednisolone pulse in the control of rheumatoid arthritis flare-up: a preliminary study. *International journal of rheumatic diseases.* 2014;17(4):389-393.
177. Salesi M, Sadeghihaddadzavareh S, Nasri P, Namdarigharaghani N, Farajzadegan Z, Hajalikhani M. The role of bromocriptine in the treatment of patients with active rheumatoid arthritis. *International journal of rheumatic diseases.* 2013;16(6):662-666.
178. Scheerens H, Su Z, Irving B, et al. MTRX1011A, a humanized anti-CD4 monoclonal antibody, in the treatment of patients with rheumatoid arthritis: a phase I randomized, double-blind, placebo-controlled study incorporating pharmacodynamic biomarker assessments. *Arthritis research & therapy.* 2011;13(5):R177.
179. Schiff M, Keiserman M, Codding C, et al. Efficacy and safety of abatacept or infliximab vs placebo in ATTEST: a phase III, multi-centre, randomised, double-blind, placebo-controlled study in patients with rheumatoid arthritis and an inadequate response to methotrexate. *Annals of the rheumatic diseases.* 2008;67(8):1096-1103.
180. Schiff MH, von Kempis J, Goldblum R, Tesser JR, Mueller RB. Rheumatoid arthritis secondary non-responders to TNF can attain an efficacious and safe response by switching to certolizumab pegol: a phase IV, randomised, multicentre, double-blind, 12-week study, followed by a 12-week open-label phase. *Annals of the rheumatic diseases.* 2014;73(12):2174-2177.
181. Senolt L, Leszczynski P, Dokoupilova E, et al. Efficacy and Safety of Anti-Interleukin-20 Monoclonal Antibody in Patients with Rheumatoid Arthritis: A Randomized Phase IIa Trial. *Arthritis & rheumatology (Hoboken, NJ).* 2015;67(6):1438-1448.
182. Seymour M, Petavy F, Chiesa F, et al. Ultrasonographic measures of synovitis in an early phase clinical trial: a double-blind, randomised, placebo and comparator-controlled phase IIa trial of GW274150 (a selective inducible nitric oxide synthase inhibitor) in rheumatoid arthritis. *Clinical and experimental rheumatology.* 2012;30(2):254-261.
183. Seymour MW, Kelly S, Beals CR, et al. Ultrasound of metacarpophalangeal joints is a sensitive and reliable endpoint for drug therapies in rheumatoid arthritis: results of a randomized, two-center placebo-controlled study. *Arthritis research & therapy.* 2012;14(5):R198.
184. Smolen JS, Agarwal SK, Ilivanova E, et al. A randomised phase II study evaluating the efficacy and safety of subcutaneously administered ustekinumab and guselkumab in patients with active rheumatoid arthritis despite treatment with methotrexate. *Annals of the rheumatic diseases.* 2017;76(5):831-839.
185. Smolen JS, Burmester GR, Combe B, et al. Head-to-head comparison of certolizumab pegol versus adalimumab in rheumatoid arthritis: 2-year efficacy and safety results from the randomised EXXELERATE study. *Lancet (London, England).* 2016;388(10061):2763-2774.
186. Smolen JS, Cohen SB, Tony HP, et al. A randomised, double-blind trial to demonstrate bioequivalence of GP2013 and reference rituximab combined with methotrexate in patients with active rheumatoid arthritis. *Annals of the rheumatic diseases.* 2017;76(9):1598-1602.

© 2019 Strait A et al. *JAMA Network Open.*

187. Smolen JS, Kay J, Doyle MK, et al. Golimumab in patients with active rheumatoid arthritis after treatment with tumour necrosis factor alpha inhibitors (GO-AFTER study): a multicentre, randomised, double-blind, placebo-controlled, phase III trial. *Lancet (London, England).* 2009;374(9685):210-221.
188. Smolen JS, Nash P, Durez P, et al. Maintenance, reduction, or withdrawal of etanercept after treatment with etanercept and methotrexate in patients with moderate rheumatoid arthritis (PRESERVE): a randomised controlled trial. *Lancet (London, England).* 2013;381(9870):918-929.
189. Smolen JS, Weinblatt ME, Sheng S, Zhuang Y, Hsu B. Sirukumab, a human anti-interleukin-6 monoclonal antibody: a randomised, 2-part (proof-of-concept and dose-finding), phase II study in patients with active rheumatoid arthritis despite methotrexate therapy. *Annals of the rheumatic diseases.* 2014;73(9):1616-1625.
190. Stock TC, Bloom BJ, Wei N, et al. Efficacy and safety of CE-224,535, an antagonist of P2X7 receptor, in treatment of patients with rheumatoid arthritis inadequately controlled by methotrexate. *The Journal of rheumatology.* 2012;39(4):720-727.
191. Stohl W, Gomez-Reino J, Olech E, et al. Safety and efficacy of ocrelizumab in combination with methotrexate in MTX-naive subjects with rheumatoid arthritis: the phase III FILM trial. *Annals of the rheumatic diseases.* 2012;71(8):1289-1296.
192. Stohl W, Merrill JT, McKay JD, et al. Efficacy and safety of belimumab in patients with rheumatoid arthritis: a phase II, randomized, double-blind, placebo-controlled, dose-ranging Study. *The Journal of rheumatology.* 2013;40(5):579-589.
193. Tak PP, Balanescu A, Tseluyko V, et al. Chemokine receptor CCR1 antagonist CCX354-C treatment for rheumatoid arthritis: CARAT-2, a randomised, placebo controlled clinical trial. *Annals of the rheumatic diseases.* 2013;72(3):337-344.
194. Tak PP, Rigby WF, Rubbert-Roth A, et al. Inhibition of joint damage and improved clinical outcomes with rituximab plus methotrexate in early active rheumatoid arthritis: the IMAGE trial. *Annals of the rheumatic diseases.* 2011;70(1):39-46.
195. Tak PP, Thurlings RM, Rossier C, et al. Atacicept in patients with rheumatoid arthritis: results of a multicenter, phase Ib, double-blind, placebo-controlled, dose-escalating, single- and repeated-dose study. *Arthritis and rheumatism.* 2008;58(1):61-72.
196. Takeuchi T, Harigai M, Tanaka Y, et al. Golimumab monotherapy in Japanese patients with active rheumatoid arthritis despite prior treatment with disease-modifying antirheumatic drugs: results of the phase 2/3, multicentre, randomised, double-blind, placebo-controlled GO-MONO study through 24 weeks. *Annals of the rheumatic diseases.* 2013;72(9):1488-1495.
197. Takeuchi T, Miyasaka N, Inoue K, Abe T, Koike T. Impact of trough serum level on radiographic and clinical response to infliximab plus methotrexate in patients with rheumatoid arthritis: results from the RISING study. *Modern rheumatology.* 2009;19(5):478-487.
198. Takeuchi T, Miyasaka N, Zang C, et al. A phase 3 randomized, double-blind, multicenter comparative study evaluating the effect of etanercept versus methotrexate on radiographic outcomes, disease activity, and safety in Japanese subjects with active rheumatoid arthritis. *Modern rheumatology.* 2013;23(4):623-633.
199. Takeuchi T, Tanaka Y, Ishiguro N, et al. Effect of denosumab on Japanese patients with rheumatoid arthritis: a dose-response study of AMG 162 (Denosumab) in patients with RheumatoId arthritis on methotrexate to Validate inhibitory effect on bone Erosion (DRIVE)-a 12-month, multicentre, randomised, double-blind, placebo-controlled, phase II clinical trial. *Annals of the rheumatic diseases.* 2016;75(6):983-990.
200. Takeuchi T, Tanaka Y, Iwasaki M, Ishikura H, Saeki S, Kaneko Y. Efficacy and safety of the oral Janus kinase inhibitor peficitinib (ASP015K) monotherapy in patients with moderate to severe rheumatoid arthritis in Japan: a 12-week, randomised, double-blind, placebo-controlled phase IIb study. *Annals of the rheumatic diseases.* 2016;75(6):1057-1064.
201. Takeuchi T, Tanaka Y, Yamanaka H, et al. Efficacy and safety of olokizumab in Asian patients with moderate-to-severe rheumatoid arthritis, previously exposed to anti-TNF therapy: Results from a randomized phase II trial. *Modern rheumatology.* 2016;26(1):15-23.
202. Takeuchi T, Yamanaka H, Ishiguro N, et al. Adalimumab, a human anti-TNF monoclonal antibody, outcome study for the prevention of joint damage in Japanese patients with early rheumatoid arthritis: the HOPEFUL 1 study. *Annals of the rheumatic diseases.* 2014;73(3):536-543.
203. Tanaka Y, Emoto K, Cai Z, et al. Efficacy and Safety of Baricitinib in Japanese Patients with Active Rheumatoid Arthritis Receiving Background Methotrexate Therapy: A 12-week, Double-blind, Randomized Placebo-controlled Study. *The Journal of rheumatology.* 2016;43(3):504-511.
204. Tanaka Y, Harigai M, Takeuchi T, et al. Golimumab in combination with methotrexate in Japanese patients with active rheumatoid arthritis: results of the GO-FORTH study. *Annals of the rheumatic diseases.* 2012;71(6):817-824.

© 2019 Strait A et al. *JAMA Network Open.*

205. Tanaka Y, Takeuchi T, Yamanaka H, Nakamura H, Toyoizumi S, Zwillich S. Efficacy and safety of tofacitinib as monotherapy in Japanese patients with active rheumatoid arthritis: a 12-week, randomized, phase 2 study. *Modern rheumatology.* 2015;25(4):514-521.
206. Taylor PC, Genovese MC, Greenwood M, et al. OSKIRA-4: a phase IIb randomised, placebo-controlled study of the efficacy and safety of fostamatinib monotherapy. *Annals of the rheumatic diseases.* 2015;74(12):2123-2129.
207. Taylor PC, Keystone EC, van der Heijde D, et al. Baricitinib versus Placebo or Adalimumab in Rheumatoid Arthritis. *The New England journal of medicine.* 2017;376(7):652-662.
208. Taylor PC, Quattrocchi E, Mallett S, Kurrasch R, Petersen J, Chang DJ. Ofatumumab, a fully human anti-CD20 monoclonal antibody, in biological-naive, rheumatoid arthritis patients with an inadequate response to methotrexate: a randomised, double-blind, placebo-controlled clinical trial. *Annals of the rheumatic diseases.* 2011;70(12):2119-2125.
209. Tlustochowicz W, Rahman P, Seriolo B, et al. Efficacy and Safety of Subcutaneous and Intravenous Loading Dose Regimens of Secukinumab in Patients with Active Rheumatoid Arthritis: Results from a Randomized Phase II Study. *The Journal of rheumatology.* 2016;43(3):495-503.
210. van der Heijde D, Tanaka Y, Fleischmann R, et al. Tofacitinib (CP-690,550) in patients with rheumatoid arthritis receiving methotrexate: twelve-month data from a twenty-four-month phase III randomized radiographic study. *Arthritis and rheumatism.* 2013;65(3):559-570.
211. van Kuijk AW, Vergunst CE, Gerlag DM, et al. CCR5 blockade in rheumatoid arthritis: a randomised, double-blind, placebo-controlled clinical trial. *Annals of the rheumatic diseases.* 2010;69(11):2013-2016.
212. van Vollenhoven RF, Fleischmann R, Cohen S, et al. Tofacitinib or adalimumab versus placebo in rheumatoid arthritis. *The New England journal of medicine.* 2012;367(6):508-519.
213. van Vollenhoven RF, Houbiers JG, Buttgereit F, et al. The selective estrogen receptor alpha agonist Org 37663 induces estrogenic effects but lacks antirheumatic activity: a phase IIa trial investigating efficacy and safety of Org 37663 in postmenopausal female rheumatoid arthritis patients receiving stable background methotrexate or sulfasalazine. *Arthritis and rheumatism.* 2010;62(2):351-358.
214. van Vollenhoven RF, Kinnman N, Vincent E, Wax S, Bathon J. Atacicept in patients with rheumatoid arthritis and an inadequate response to methotrexate: results of a phase II, randomized, placebo-controlled trial. *Arthritis and rheumatism.* 2011;63(7):1782-1792.
215. van Vollenhoven RF, Ostergaard M, Leirisalo-Repo M, et al. Full dose, reduced dose or discontinuation of etanercept in rheumatoid arthritis. *Annals of the rheumatic diseases.* 2016;75(1):52-58.
216. van Vollenhoven RF, Wax S, Li Y, Tak PP. Safety and efficacy of atacicept in combination with rituximab for reducing the signs and symptoms of rheumatoid arthritis: a phase II, randomized, double-blind, placebo-controlled pilot trial. *Arthritis & rheumatology (Hoboken, NJ).* 2015;67(11):2828-2836.
217. Vergunst CE, Gerlag DM, Lopatinskaya L, et al. Modulation of CCR2 in rheumatoid arthritis: a double-blind, randomized, placebo-controlled clinical trial. *Arthritis and rheumatism.* 2008;58(7):1931-1939.
218. Vergunst CE, Gerlag DM, von Moltke L, et al. MLN3897 plus methotrexate in patients with rheumatoid arthritis: safety, efficacy, pharmacokinetics, and pharmacodynamics of an oral CCR1 antagonist in a phase IIa, double-blind, placebo-controlled, randomized, proof-of-concept study. *Arthritis and rheumatism.* 2009;60(12):3572-3581.
219. Vital EM, Dass S, Buch MH, Rawstron AC, Emery P. An extra dose of rituximab improves clinical response in rheumatoid arthritis patients with initial incomplete B cell depletion: a randomised controlled trial. *Annals of the rheumatic diseases.* 2015;74(6):1195-1201.
220. Weinblatt ME, Bingham CO, 3rd, Mendelsohn AM, et al. Intravenous golimumab is effective in patients with active rheumatoid arthritis despite methotrexate therapy with responses as early as week 2: results of the phase 3, randomised, multicentre, double-blind, placebo-controlled GO-FURTHER trial. *Annals of the rheumatic diseases.* 2013;72(3):381-389.
221. Weinblatt ME, Fleischmann R, Huizinga TW, et al. Efficacy and safety of certolizumab pegol in a broad population of patients with active rheumatoid arthritis: results from the REALISTIC phase IIIb study. *Rheumatology (Oxford, England).* 2012;51(12):2204-2214.
222. Weinblatt ME, Genovese MC, Ho M, et al. Effects of fostamatinib, an oral spleen tyrosine kinase inhibitor, in rheumatoid arthritis patients with an inadequate response to methotrexate: results from a phase III, multicenter, randomized, double-blind, placebo-controlled, parallel-group study. *Arthritis & rheumatology (Hoboken, NJ).* 2014;66(12):3255-3264.
223. Weinblatt ME, Kavanaugh A, Burgos-Vargas R, et al. Treatment of rheumatoid arthritis with a Syk kinase inhibitor: a twelve-week, randomized, placebo-controlled trial. *Arthritis and rheumatism.* 2008;58(11):3309-3318.
224. Weinblatt ME, Kavanaugh A, Genovese MC, Musser TK, Grossbard EB, Magilavy DB. An oral spleen tyrosine kinase (Syk) inhibitor for rheumatoid arthritis. *The New England journal of medicine.* 2010;363(14):1303-1312.

© 2019 Strait A et al. *JAMA Network Open.*

225. Weinblatt ME, Mease P, Mysler E, et al. The efficacy and safety of subcutaneous clazakizumab in patients with moderate-to-severe rheumatoid arthritis and an inadequate response to methotrexate: results from a multinational, phase IIb, randomized, double-blind, placebo/active-controlled, dose-ranging study. *Arthritis & rheumatology (Hoboken, NJ).* 2015;67(10):2591-2600.
226. Weinblatt ME, Schiff MH, Ruderman EM, et al. Efficacy and safety of etanercept 50 mg twice a week in patients with rheumatoid arthritis who had a suboptimal response to etanercept 50 mg once a week: results of a multicenter, randomized, double-blind, active drug-controlled study. *Arthritis and rheumatism.* 2008;58(7):1921-1930.
227. Westhovens R, Keyser FD, Rekalov D, et al. Oral administration of GLPG0259, an inhibitor of MAPKAPK5, a new target for the treatment of rheumatoid arthritis: a phase II, randomised, double-blind, placebo-controlled, multicentre trial. *Annals of the rheumatic diseases.* 2013;72(5):741-744.
228. Westhovens R, Robles M, Ximenes AC, et al. Clinical efficacy and safety of abatacept in methotrexate-naive patients with early rheumatoid arthritis and poor prognostic factors. *Annals of the rheumatic diseases.* 2009;68(12):1870-1877.
229. Westhovens R, Taylor PC, Alten R, et al. Filgotinib (GLPG0634/GS-6034), an oral JAK1 selective inhibitor, is effective in combination with methotrexate (MTX) in patients with active rheumatoid arthritis and insufficient response to MTX: results from a randomised, dose-finding study (DARWIN 1). *Annals of the rheumatic diseases.* 2017;76(6):998-1008.
230. Wijesinghe H, Galappatthy P, de Silva R, et al. Leflunomide is equally efficacious and safe compared to low dose rituximab in refractory rheumatoid arthritis given in combination with methotrexate: results from a randomized double blind controlled clinical trial. *BMC musculoskeletal disorders.* 2017;18(1):310.
231. Winthrop KL, Wouters AG, Choy EH, et al. The Safety and Immunogenicity of Live Zoster Vaccination in Patients with Rheumatoid Arthritis Before Starting Tofacitinib: A Randomized Phase II Trial. *Arthritis & rheumatology (Hoboken, NJ).* 2017;69(10):1969-1977.
232. Wisniacki N, Amaravadi L, Galluppi GR, et al. Safety, tolerability, pharmacokinetics, and pharmacodynamics of anti-TWEAK monoclonal antibody in patients with rheumatoid arthritis. *Clinical therapeutics.* 2013;35(8):1137-1149.
233. Wong M, Oakley SP, Young L, et al. Infliximab improves vascular stiffness in patients with rheumatoid arthritis. *Annals of the rheumatic diseases.* 2009;68(8):1277-1284.
234. Yamamoto K, Takeuchi T, Yamanaka H, et al. Efficacy and safety of certolizumab pegol without methotrexate co-administration in Japanese patients with active rheumatoid arthritis: the HIKARI randomized, placebo-controlled trial. *Modern rheumatology.* 2014;24(4):552-560.
235. Yamamoto K, Takeuchi T, Yamanaka H, et al. Efficacy and safety of certolizumab pegol plus methotrexate in Japanese rheumatoid arthritis patients with an inadequate response to methotrexate: the J-RAPID randomized, placebo-controlled trial. *Modern rheumatology.* 2014;24(5):715-724.
236. Yazici Y, Curtis JR, Ince A, et al. Efficacy of tocilizumab in patients with moderate to severe active rheumatoid arthritis and a previous inadequate response to disease-modifying antirheumatic drugs: the ROSE study. *Annals of the rheumatic diseases.* 2012;71(2):198-205.
237. Yellin M, Paliienko I, Balanescu A, et al. A phase II, randomized, double-blind, placebo-controlled study evaluating the efficacy and safety of MDX-1100, a fully human anti-CXCL10 monoclonal antibody, in combination with methotrexate in patients with rheumatoid arthritis. *Arthritis and rheumatism.* 2012;64(6):1730-1739.
238. Yoo DH, Hrycaj P, Miranda P, et al. A randomised, double-blind, parallel-group study to demonstrate equivalence in efficacy and safety of CT-P13 compared with innovator infliximab when coadministered with methotrexate in patients with active rheumatoid arthritis: the PLANETRA study. *Annals of the rheumatic diseases.* 2013;72(10):1613-1620.
239. Yoo DH, Suh CH, Shim SC, et al. A multicentre randomised controlled trial to compare the pharmacokinetics, efficacy and safety of CT-P10 and innovator rituximab in patients with rheumatoid arthritis. *Annals of the rheumatic diseases.* 2017;76(3):566-570.
240. Zhang W, Kernstock RM, Karrer EE, et al. A Phase 1 Dose-Escalation Study of ASP2409, a Selective T-Cell Costimulation Inhibitor, in Stable Rheumatoid Arthritis Patients on Methotrexate Therapy. *Clinical pharmacology in drug development.* 2016;5(4):259-268.

© 2019 Strait A et al. *JAMA Network Open.*
